# A defined glycosylation regulatory network modulates total glycome dynamics during pluripotency state transition

**DOI:** 10.1038/s41598-020-79666-4

**Published:** 2021-01-14

**Authors:** Federico Pecori, Ikuko Yokota, Hisatoshi Hanamatsu, Taichi Miura, Chika Ogura, Hayato Ota, Jun-ichi Furukawa, Shinya Oki, Kazuo Yamamoto, Osamu Yoshie, Shoko Nishihara

**Affiliations:** 1 Laboratory of Cell Biology, Department of Bioinformatics, Graduate School of Engineering, Soka University, 1-236 Tangi-machi, Hachioji, Tokyo 192-8577 Japan; 2 Department of Advanced Clinical Glycobiology, Faculty of Medicine and Graduate School of Medicine, Hokkaido University, Kita 15, Nishi 7, Kita-ku, Sapporo, Hokkaido 060-8638 Japan; 3 Department of Drug Discovery Medicine, Graduate School of Medicine, Kyoto University, 53 Shogoin Kawahara-cho, Sakyo-ku, Kyoto, 606-8507 Japan; 4 Department of Integrated Biosciences, Graduate School of Frontier Sciences, The University of Tokyo, 5-1-5 Kashiwanoha, Kashiwa, Chiba 277-8562 Japan; 5 Health and Kampo Institute, 1-11-10 Murasakiyama, Izumi, Sendai, Miyagi 981-3205 Japan; 6 Glycan and Life System Integration Center (GaLSIC), Faculty of Science and Engineering, Soka University, 1-236 Tangi-machi, Hachioji, Tokyo 192-8577 Japan; 7 Present Address: National Institute of Radiological Sciences (NIRS), National Institutes for Quantum and Radiological Science and Technology, 4-9-1 Anagawa, Inage-ku, Chiba, 263-8555 Japan

**Keywords:** Glycosylation, Glycobiology, Glycomics, Pluripotency, Pluripotent stem cells, Epigenetics

## Abstract

Embryonic stem cells (ESCs) and epiblast-like cells (EpiLCs) recapitulate in vitro the epiblast first cell lineage decision, allowing characterization of the molecular mechanisms underlying pluripotent state transition. Here, we performed a comprehensive and comparative analysis of total glycomes of mouse ESCs and EpiLCs, revealing that overall glycosylation undergoes dramatic changes from early stages of development. Remarkably, we showed for the first time the presence of a developmentally regulated network orchestrating glycosylation changes and identified polycomb repressive complex 2 (PRC2) as a key component involved in this process. Collectively, our findings provide novel insights into the naïve-to-primed pluripotent state transition and advance the understanding of glycosylation complex regulation during early mouse embryonic development.

## Introduction

Mammalian embryonic development is a complex stepwise process. Following cleavage and compaction, the embryo organizes itself into two layers: the inner cell mass (ICM) and the trophoblast cells. At this stage, the first ICM commitment occurs. The segregation of cells within the ICM forms the epiblast (EPI) and the primitive endoderm. Cells generating the EPI can differentiate into all embryonic tissues and its associated extraembryonic tissues, hence are pluripotent stem cells (PSCs). After segregation, the embryo implants into the uterine epithelium, initiating significant alterations in stem cells identity and embryo morphology^1^. Embryonic stem cells (ESCs) are derived from pre-implantation embryos at E3.5-E4.5^2^. EPI-like cells (EpiLCs), which differentiate from ESCs in culture, resemble the post-implantation stage at E5.5-E6.5^3^. ESCs and EpiLCs reflect two distinct pluripotent states known as the naïve state and the primed state, respectively, providing a useful model system to examine the pluripotent state transition occurring at implantation in vitro^4^. PSCs represent a promising tool to dissect the mechanisms underpinning mammalian development and advance regenerative medicine. Indeed, a large number of studies have investigated the changes underlying the transition from pre- to post-implantation stage both in vivo and in vitro^5–7^. However, a significant amount of attributes still remain unexplored, hampering ESC exploitation.

Glycosylation, which is expected to be present on more than half of all mammalian proteins^8^, is one of the most abundant post-translational modifications and is exerted by over 200 distinct glycosyltransferases and related enzymes. Glycosylation is involved in a wide range of cellular processes, such as adhesion, signaling regulation, endocytosis, protein folding and protein stability^9^. To date, numerous studies have reported the critical role of glycosylation in development and stem cell regulation across different species^10,11^. For instance, mutation of *C1GalT1*, a key enzyme of the mucin-type *O*-glycosylation pathway, causes embryonic lethality in mice and alters the localization of neuromuscular junctions and establishment of muscle cell architecture in *Drosophila*^12,13^. Heparan sulfate (HS) reduction leads to differentiation of mouse ESCs and *Drosophila* germline stem cells^14,15^. Moreover, *O*-linked *N*-acetylglucosamine transferase (Ogt) has been shown to be essential for embryogenesis and early development in several animal models^16,17^, and for maintenance of the naïve state in mouse ESCs^18,19^. The overall glycomic profile has been characterized in mouse ESCs, conventional human ESCs (hESCs) and human induced pluripotent stem cells (hiPSCs), which are in a primed state, tumors and late differentiating cells^20–22^. However, a comparative analysis of glycomes during mammalian early embryonic development is currently missing.

Here, we performed a comprehensive and comparative analysis of the glycome of mouse ESCs and EpiLCs. Our findings show that all glycosylation classes undergo dramatic changes, both at the transcriptional and the structural level, from early stages of development. Furthermore, we identified polycomb repressive complex 2 (PRC2), a chromatin-remodeling complex which deposits three methyl groups at histone H3 lysine 27 (H3K27me3) to promote gene repression^23,24^, as one key component involved in the network orchestrating these glycosylation changes. Our results provide direct insights into glycosylation dynamics and contribute to the understanding of glycosylation regulation during mouse early embryonic development.

## Results

### Induction of EpiLCs from ESCs

**Figure 1:**
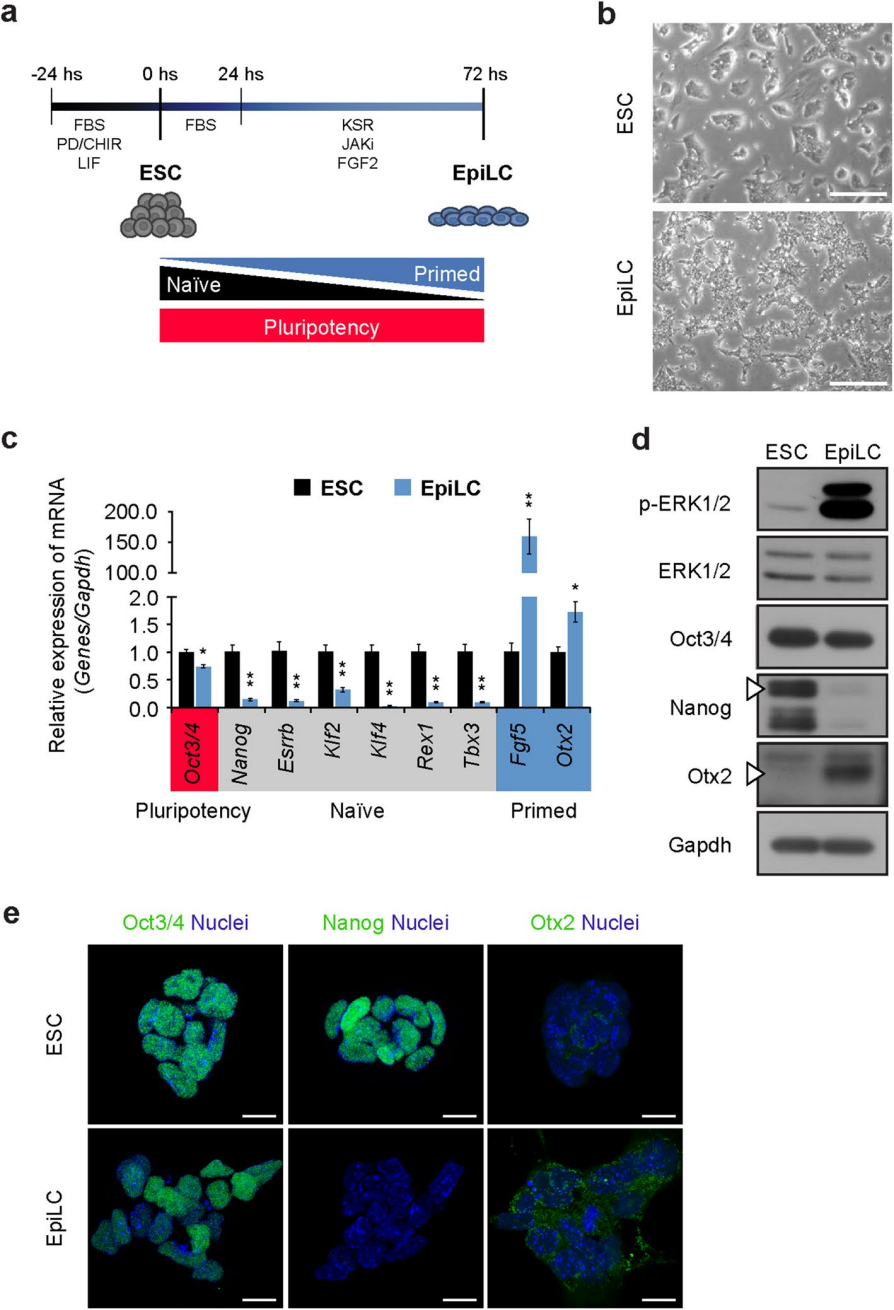
EpiLC differentiation from ESCs. (**a**) Schematic representation of EpiLC differentiation protocol from ESCs. (**b**) Morphology of ESCs (upper panel), and EpiLCs (lower panel). Scale bar, 200 μm. (**c**) Real-time PCR analysis of pluripotency (red), naïve (grey), and primed (blue) markers normalized against *Gapdh* in ESCs and EpiLCs, and shown as a fold change relative to ESCs. (**d**) Representative cropped image of a western blot analysis of p-ERK1/2, ERK1/2, Oct3/4, Nanog, Otx2 and Gapdh in ESCs and EpiLCs. Arrowheads indicate the specific protein bands. Full-length blots are presented in Supplementary Fig. S15. (**e**) Representative image of permeabilized ESCs and EpiLCs after immunostaining using anti-Oct3/4, anti-Nanog and anti-Otx2 Abs. Nuclei were stained with Hoechst. Scale bar, 10 μm. Values are shown as means ± s.e.m. of three independent experiments. Significant values are indicated as *P < 0.05, and **P < 0.01.

To investigate the glycosylation dynamics occurring at the implantation stage, we induced differentiation of EpiLCs from ESCs. ESCs and EpiLCs are dependent on leukemia inhibitory factor (LIF) and fibroblast growth factor (FGF) signaling, respectively^25,26^. Here, we used a previously established protocol^3^ with slight modifications (Fig. 1a). EpiLCs exhibit a typical flattened morphology after 72 h of induction (Fig. 1b). Transcriptional analysis of EpiLCs by Real-time PCR showed a negligible change in the pluripotency marker *Oct3/4*, whereas genes associated with the naïve state, such as *Nanog, Esrrb, Klf2, Klf4, Rex1* and *Tbx3* were strongly downregulated, together with a striking increase in the primed markers *Fgf5*, and *Otx2* (Fig. 1c)*,* consistently with previous results^3, 27^. Accordingly, Oct3/4 protein level was retained in EpiLC at levels similar to those in ESCs, whereas the naïve marker Nanog decreased and the primed marker Otx2 increased. Furthermore, the phosphorylation level of ERK1/2, a downstream kinase involved in FGF signaling^26^, was substantially higher in EpiLCs (Fig. 1d). The expression of these markers was further assessed by immunostaining: Oct3/4 staining slightly decreased during the transition from ESCs to EpiLCs, whereas Nanog was not detected and Otx2 was highly expressed in EpiLCs, further confirming the successful differentiation of EpiLCs from ESCs (Fig. 1e).

### Comprehensive and comparative glycome profiling of ESCs and EpiLCs *N*-linked glycosylation and free oligosaccharides

**Figure 2:**
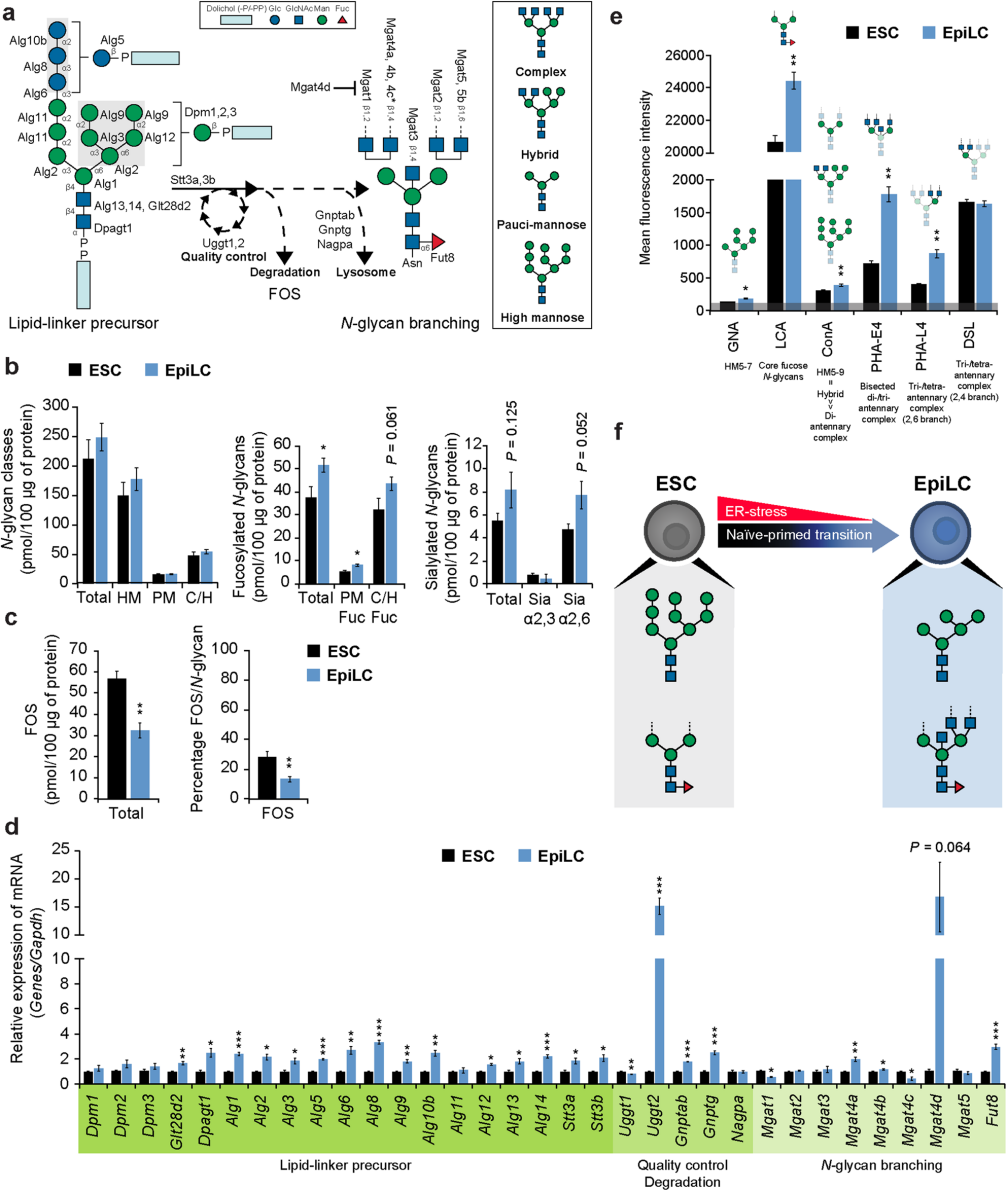
ESC and EpiLC *N*-glycosylation structural and transcriptional analysis. (**a**) Schematic diagram of the *N*-glycosylation pathway. The asterisk denotes enzyme putative activity. (**b**) Absolute amount of *N*-glycans detected by MS in ESCs and EpiLCs. *N*-glycosylation subclasses: high mannose-type (HM), pauci-mannose (PM), and complex/hybrid (C/H). Fucose: Fuc; sialylation: Sia. (**c**) Absolute amount of FOS detected by MS in ESCs and EpiLCs (left panel) and percentage of FOS relative to total amount of *N*-glycans (right panel). (**d**) Real-time PCR analysis of *N*-glycosylation-specific enzymes normalized against *Gapdh* in ESCs and EpiLCs, and shown as a fold change relative to ESCs. (**e**) *N*-glycan structures profiling by FACS using specific lectins in ESCs and EpiLCs. Lectins specificities are stated below the labels and schematically represented above each histogram. The grey line at the bottom represents the negative control staining. (**f**) Model of *N*-glycome alterations during ESC to EpiLC transition. Values are shown as means ± s.e.m. of three independent experiments in (**d**,**e**) and four independent experiments in (**b**,**c**). Significant values are indicated as *P < 0.05, **P < 0.01, and ***P < 0.001.

*N*-linked glycosylation involves the transfer of a tetradecasaccharide core unit from a lipid-linker donor to an asparagine of nascent proteins at the endoplasmic reticulum (ER). Upon transfer to the polypeptide chain, the glycoprotein undergoes trimming and a quality control process to ensure its correct folding. Fully folded glycoproteins are transported to the Golgi where they are subjected to further trimming and processing prior to transportation to the plasma membrane, whereas misfolded proteins are recycled, resulting in the release of free oligosaccharides (FOS)^28,29^ (Fig. 2a). *N*-glycans and FOS composition were quantified by mass spectrometry (MS) analysis. The total amount of *N*-glycans was similar between ESCs and EpiLCs. Among the detected *N*-glycan subclasses, namely high mannose-type (HM), pauci-mannose (PM), and complex/hybrid (C/H), HM structures were the most abundant of the detected *N*-glycans in both ESCs and EpiLCs, confirming previous results obtained in conventional hESCs and hiPSCs^21^. Fucosylation and sialylation of *N*-glycans diverged dramatically between ESCs and EpiLCs. The total amount of fucosylated *N*-glycans was higher in EpiLCs compared to ESCs and was characterized by increased levels of both fucosylated PM and C/H structures. Total sialylated *N*-glycans, mainly present in an α2,6 configuration, were enhanced in EpiLCs (Fig. 2b and Supplementary Fig. S1). FOS amount reportedly increases under conditions of ER stress^29^. A sharp reduction in the total amount and relative percentage of FOS was observed in EpiLCs, indicating a reduction of ER stress upon transition from ESC to EpiLC (Fig. 2c and Supplementary Fig. S2). Interestingly, a recent report demonstrated that ER stress positively modulates interleukin-6 family expression (including LIF) in mouse astrocytes and macrophages^30^, suggesting a connection between FOS, ER stress, and LIF expression in ESCs. Transcriptional analysis of *N*-glycosylation pathway-specific genes well correlated with MS data. Indeed, *Fut8*, the sole enzyme responsible for catalyzing *N*-glycans core fucosylation^31^, showed an increased expression. Interestingly, a robust enhancement of *Uggt2* was observed in EpiLCs, suggesting that a reduction in FOS-mediated ER-stress is partially mediated by increased expression of enzymes involved in *N*-glycans quality control and folding (Fig. 2d). To obtain more detailed insights into the *N*-glycome we performed a FACS profiling using a set of lectins that recognize *N*-glycan structures with different specificity. As a result, we observed a shift to shorter HM by *galanthus nivalis* agglutinin (GNA). Moreover, the increase in core fucosylated *N*-glycans observed at the structural and transcriptional level in EpiLCs was further confirmed by *lens culinaris* agglutinin (LCA) staining. In addition, *phaseolus vulgaris* erythroagglutinin (PHA-E4) and *phaseolus vulgaris* leucoagglutinin (PHA-L4) profiling indicated an enhancement in *N*-glycans carrying bisecting GlcNAc and 2,6-branching in EpiLCs, suggesting a relevant role for these structures during EpiLC differentiation (Fig. 2e and Supplementary Fig. S3). Together, these data demonstrate that the *N*-glycome composition greatly diverges between ESCs and EpiLCs. The increase in short HM structures, shown by GNA staining (Fig. 2e), and the core fucosylated *N*-glycans carrying bisecting GlcNAc and 2,6-branching, indicated by the increase of fucosylated PM (Fig. 2b) and by LCA, PHA-E4, and PHA-L4 staining (Fig. 2e), are more characteristic of EpiLCs *N*-glycome (Fig. 2f).

### *O*-linked glycosylation

**Figure 3:**
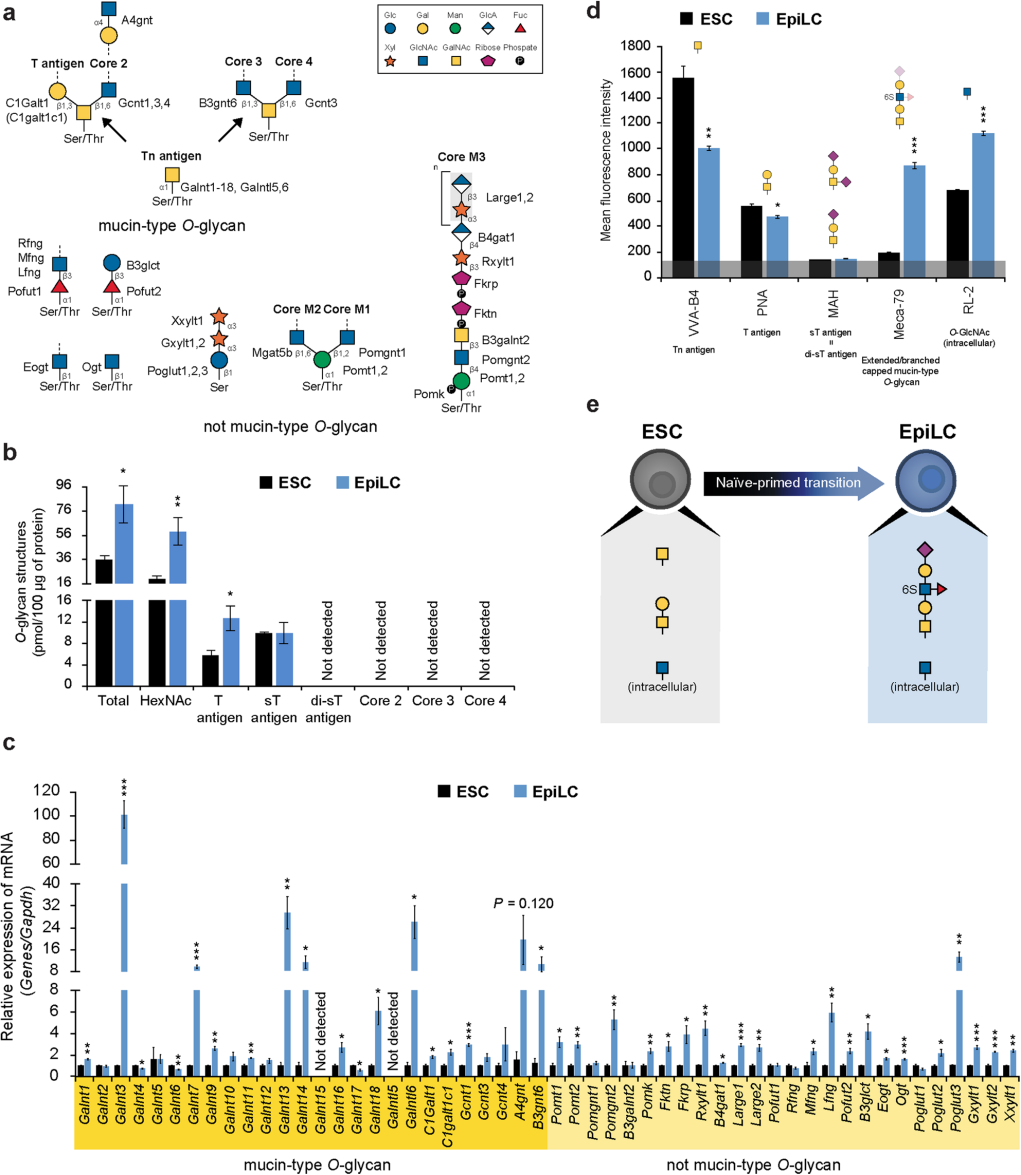
ESC and EpiLC *O*-glycosylation structural and transcriptional analysis. (**a**) Schematic diagram of the *O*-glycosylation pathway. (**b**) Absolute amount of *O*-glycans detected by MS in ESCs and EpiLCs. HexNAc: GalNAc or GlcNAc. (**c**) Real-time PCR analysis of *O*-glycosylation-specific enzymes normalized against *Gapdh* in ESCs and EpiLCs, and shown as a fold change relative to ESCs. (**d**) *O*-glycan structure profiling by FACS using specific lectins/Abs in ESCs and EpiLCs. Lectin/Ab specificities are stated below the labels and schematically represented above each histogram. The grey line at the bottom represents the negative control staining. (**e**) Model of *O*-glycome changes during ESC to EpiLC transition. Values are shown as means ± s.e.m. of three independent experiments in (**c**,**d**) and four independent experiments in (**b**). Significant values are indicated as *P < 0.05, **P < 0.01, and ***P < 0.001.

*O*-linked glycosylation is characterized by the initial addition of a monosaccharide to the hydroxyl group of a serine/threonine residue on the target protein. Various monosaccharides can be added to serine/threonine giving rise to different *O*-glycosylation subclasses: mucin-type *O*-glycosylation and not mucin-type *O*-glycosylation. Mucin-type *O*-glycosylation is initiated in the Golgi by a large family of up to 19 transferases that link *N*-acetylgalactosamine (GalNAc) on a serine/threonine residue to form Tn antigen. Tn antigen can be further elongated adopting one of 4 distinct core extensions: T antigen (Core 1), Core 2, Core 3 and Core 4^32^. Not mucin-type *O*-glycosylation takes place in the ER, except for the *O*-linked β-*N*-acetylglucosamine (*O*-GlcNAc) addition catalyzed by Ogt, the sole glycosylation that occurs in the cytoplasm and nucleus^33^ (Fig. 3a). *O*-glycome profiling by MS revealed that HexNAc (Tn antigen or *O*-GlcNAc) was the most prominent *O*-glycan structure in both ESCs and EpiLCs. Moreover, T antigen and its modified structures were the only detected *O*-glycans among the mucin-type *O*-glycosylation core structures in both ESCs and EpiLCs (Fig. 3b and Supplementary Fig. S4), indicating that the T antigen elongation pathway is the most abundant during early embryonic development, in accordance with our previous work^34^. From a transcriptional standpoint, a strong upregulation of both mucin-type *O*-glycan and not mucin-type *O*-glycan-specific genes was observed in EpiLCs, consistently with the overall enhancement of *O*-glycan content observed by MS. In particular, the expression of *Galnt3,7,13,14,18* and *Galntl6*, which are involved in the formation of Tn antigen, was dramatically increased (Fig. 3c), suggesting their major involvement in the formation of Tn antigen in EpiLCs. FACS analysis showed a shift from short mucin-type *O*-glycans, as indicated by *Vicia villosa* agglutinin-B4 (VVA-B4) and *peanut* agglutinin (PNA), which recognize Tn and T antigens, respectively, to elongated or branched mucin-type *O*-glycan structures, as shown by Meca-79 antibody (Ab) staining in EpiLCs. Intracellular *O*-GlcNAc was present in both ESCs and EpiLCs, despite it increased in EpiLCs, reflecting *Ogt* expression upregulation (Fig. 3d,e and Supplementary Fig. S5). Discrepancies in the T antigen data obtained by MS and FACS in EpiLCs may be attributed to the fact that MS detected internal glycans, hence not fully elongated, resulting in an overestimation of the total amount of T antigen.

### Glycosaminoglycans

**Figure 4:**
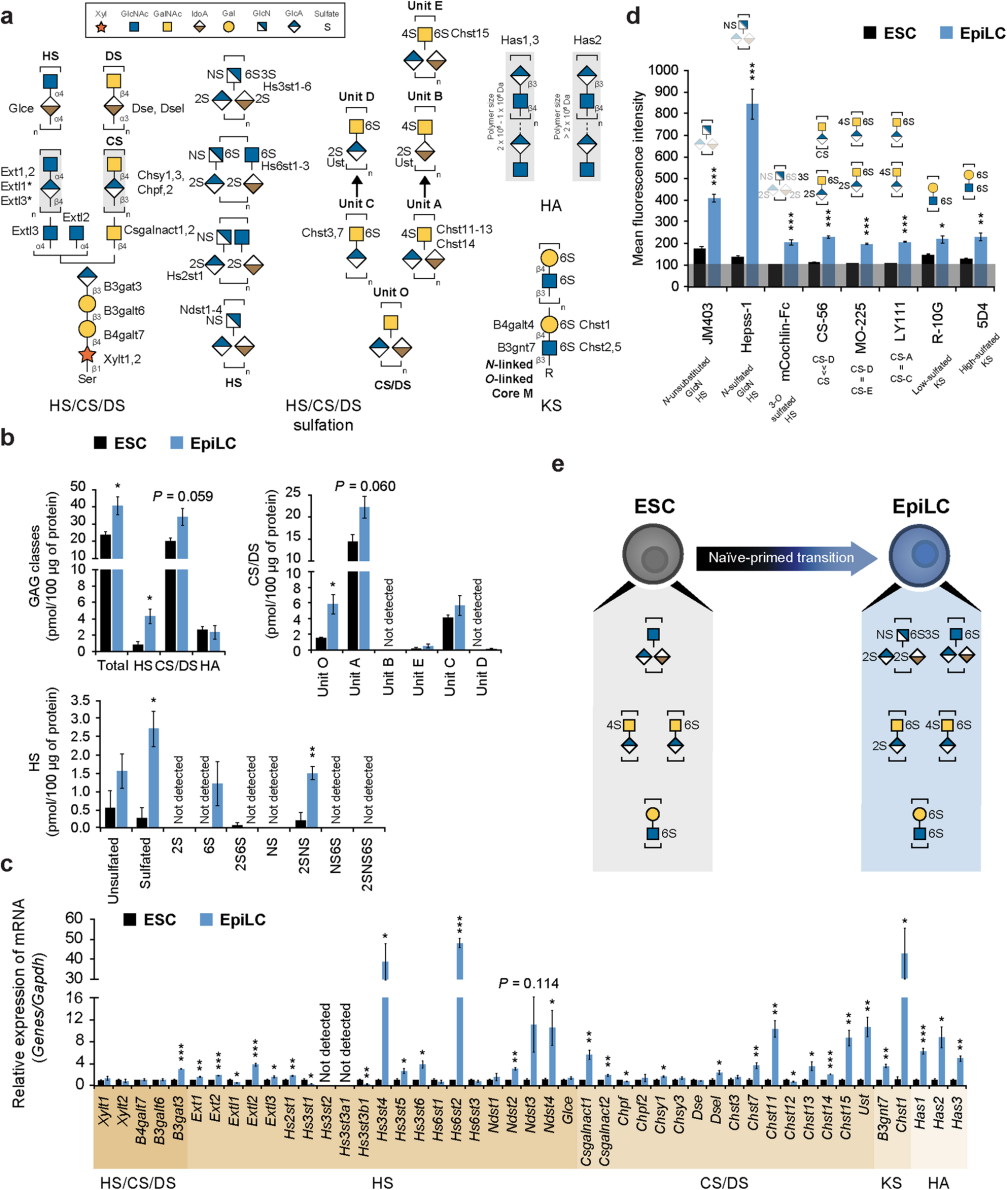
GAG structural and transcriptional analysis in ESCs and EpiLCs. (**a**) Schematic diagram of the GAG synthetic pathway. Asterisks denote enzymes whose contribution to biosynthesis remains unclear. (**b**) Absolute amount of GAG detected by HPLC in ESCs and EpiLCs. GAG subclasses: heparan sulfate (HS), chondroitin sulfate/dermatan sulfate (CS/DS) and hyaluronan (HA). (**c**) Real-time PCR analysis of GAG-specific enzymes normalized against *Gapdh* in ESCs and EpiLCs, and shown as a fold change relative to ESCs. (**d**) GAG structure profiling by FACS using specific Abs in ESCs and EpiLCs. Ab specificities are stated below the labels and schematically represented above each histogram. The grey line at the bottom represents the negative control staining. (**e**) Model of GAG modifications during ESC to EpiLC transition. Values are shown as means ± s.e.m. of three independent experiments. Significant values are indicated as *P < 0.05, **P < 0.01, and ***P < 0.001.

Glycosaminoglycans (GAG) are linear polysaccharides consisting of repeating disaccharide units attached to a core protein, resulting in proteoglycan formation. Heparan sulfate (HS), chondroitin sulfate (CS) and dermatan sulfate (DS) are connected to a serine of the core protein via a tetrasaccharide linker and are categorized by the distinct composition of their disaccharide units, sulfation and epimerization. Keratan sulfate (KS) is a sulfated polylactosamine chain extended from a GlcNAc on *N*-glycans or *O*-glycans. Conversely, unlike all the other GAG classes that are synthesized in the Golgi on core proteins, hyaluronan (HA) polymerization occurs at the cell membrane and is not linked to any protein^35^ (Fig. 4a). GAG quantification was performed by high-performance liquid chromatography (HPLC). As a result, we observed a marked increase in the total amount of GAG in EpiLCs, especially HS and CS/DS, which represent the vast majority of detected GAG, whereas HA levels did not differ between ESCs and EpiLCs; KS and 3-*O*-sulfation of HS could not be detected due to technical limitations. The absolute amount of HS sulfation was considerably higher in EpiLCs compared to ESCs, with a specific increase in mono-sulfated 6S and di-sulfated 2SNS, indicating that variations of the HS sulfation pattern occur from early developmental stages. An overall higher level of CS/DS structures was identified in EpiLCs, with predominancy of Unit A, followed by Unit C and unsulfated Unit O (Fig. 4b and Supplementary Fig. S6), suggesting a role for the 4-*O*-sulfation pathway (Unit A, B and E) during early embryonic differentiation. Transcriptional analysis of GAG-specific transferases well correlated with HPLC data. Indeed, 2SNS and 6S sulfation enhancement in EpiLCs was followed by a substantial upregulation of *Ndst2-4* and *Hs2st1*, and *Hs6st2* expression, respectively (Fig. 4c). FACS profiling confirmed the results obtained by HPLC. In addition, increased staining of di-sulfated CS, high-sulfated KS and 3-*O*-sulfated HS were observed in EpiLCs, consistently with the striking enhancement of *Ust* and *Chst15*, *Chst1*, and *Hs3st4-6* expression (Fig. 4d and Supplementary Fig. S7). The HS sulfation pattern has been shown to be critical for growth factors binding and downstream signaling. Consistently with our observations in EpiLCs, *N*-sulfation and 3-*O*-sulfation were reported to be required for exit from the naïve pluripotent state via FGF and first apoptosis signal receptor (Fas), respectively^11^. In conclusion, EpiLC GAG profile is characterized by a dramatic increase in: (i) sulfation of HS, as shown by HPLC (Fig. 4b), and by JM403, Hepss-1, and mCochlin-Fc staining (Fig. 4d); (ii) di-sulfated CS, indicated by HPLC (Unit E and D) (Fig. 4b) and by FACS using CS-56, MO-225, and LY111 (Fig. 4d); (iii) and high-sulfated KS, observed by 5D4 staining enhancement (Fig. 4d), upon differentiation from ESC (Fig. 4e).

### Glycosphingolipids

**Figure 5:**
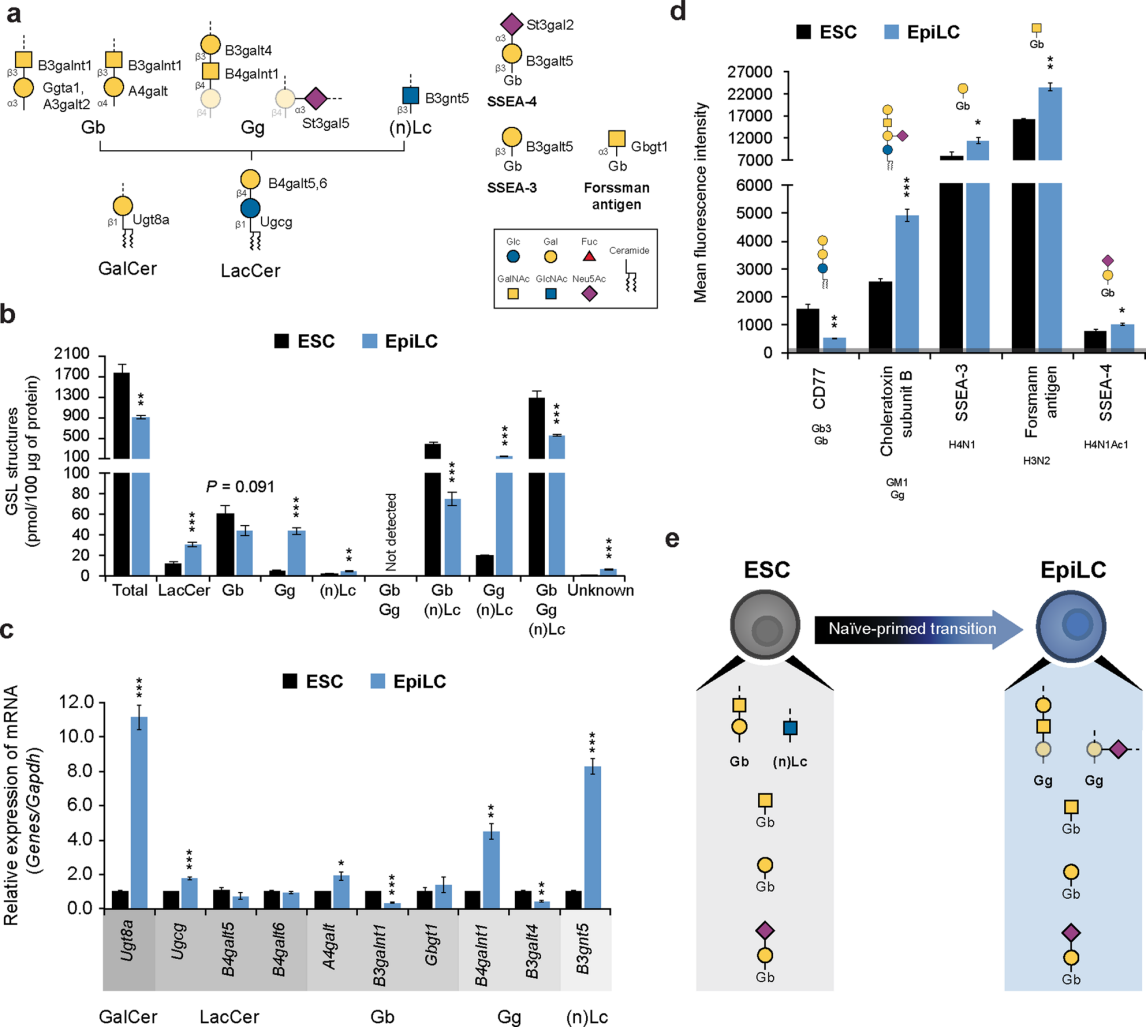
GSL structural and transcriptional analysis in ESCs and EpiLCs. (**a**) Schematic diagram of the GSL synthetic pathway. (**b**) Absolute amount of GSL detected by MS in ESCs and EpiLCs. GSL subclasses: galactosylceramide (GalCer), lactosylceramide (LacCer), globo (Gb), ganglio (Gg) and neolacto/lacto ((n)Lc). The GbGg, Gb(n)Lc, Gg(n)Lc, and GbGg(n)Lc histograms represent GSL structures which, based on the deduced composition by MS, cannot be categorize in a single subclass. (**c**) Real-time PCR analysis of GSL-specific enzymes normalized against *Gapdh* in ESCs and EpiLCs, and shown as a fold change relative to ESCs. (**d**) GSL structure profiling by FACS using specific Abs in ESCs and EpiLCs. Ab specificities are stated below the labels and schematically represented above each histogram. The grey line at the bottom represents the negative control staining. (**e**) Model of GSL modifications during ESC to EpiLC transition. Values are shown as means ± s.e.m. of three independent experiments (**c**,**d**) and four independent experiments in (**b**). Significant values are indicated as *P < 0.05, **P < 0.01, and ***P < 0.001.

Glycosphingolipids (GSL) are characterized by the initial addition of glucose or galactose to a ceramide unit to produce glucosylceramide (GlcCer) or galactosylceramide (GalCer), respectively. GlcCer synthesis occurs in the Golgi, where it is further processed to lactosylceramide (LacCer), which is the branching point for the formation of the globo (Gb), ganglio (Gg) and neolacto/lacto ((n)Lc) series. Conversely, GalCer is produced in the ER and is the precursor of the gala-series^36^ (Fig. 5a). GSL-glycan analysis was performed by glycoblotting combined with endoglycoceramidase (EGCase) I digestion. GSL composition assessed by MS showed a striking reduction of total GSL in EpiLCs. A shift from Gb and (n)Lc to Gg series was observed during ESC to EpiLC transition (Fig. 5b and Supplementary Fig. S8). GlcCer and GalCer could not be detected due to the inherent enzymatic specificity of the EGCase used to release the glycan moieties (EGCase I). Analysis of the GSL-specific genes expression showed a marked enhancement of *Ugt8a*, which is involved in the formation of GalCer, whereas LacCer formation-related transferases were mildly increased or unaltered. The Gb to Gg series switch in EpiLCs observed at the structural level was consistent also at the transcriptional level. A reduction in the expression of the Gb series-specific enzyme *B3galnt1* was followed by a robust upregulation of the Gg series-specific enzyme *B4galnt1* (Fig. 5c). Moreover, FACS analysis using specific Abs further confirmed the shift from Gb to Gg observed by MS (Fig. 5d), demonstrating that the Gb to Gg series switch previously observed in neurons and embryoid bodies derived from mouse and human ESCs, respectively^37,38^, occurs specifically during the naïve to primed pluripotent state transition. The GSL profile dynamically changes during embryonic development; as a result, specific GSL structures, such as stage-specific embryonic antigen (SSEA)-3, SSEA-4 and Forssman antigen, are used as differentiation stage markers^39^. FACS analysis showed that SSEA-3,4 and Forssman antigen staining mildly increased in EpiLCs (Fig. 5d and Supplementary Fig. S9). However, it is worth noting that SSEA-3,4 and Forssman antigen are structures of the Gb series (Fig. 5a), which we showed to be dramatically reduced and undergo a switch to Gg series upon ESC differentiation to EpiLCs, thus suggesting that Gg series structures might be more suitable differentiation markers. Together, these data show that the GSL composition shifts from Gb and (n)Lc to Gg series during ESC to EpiLC transition, and demonstrate the presence of SSEA-3,4 and Forsmann antigen structures in both ESCs and EpiLCs (Fig. 5e).

### Elongation/branching and capping/terminal modifications

**Figure 6:**
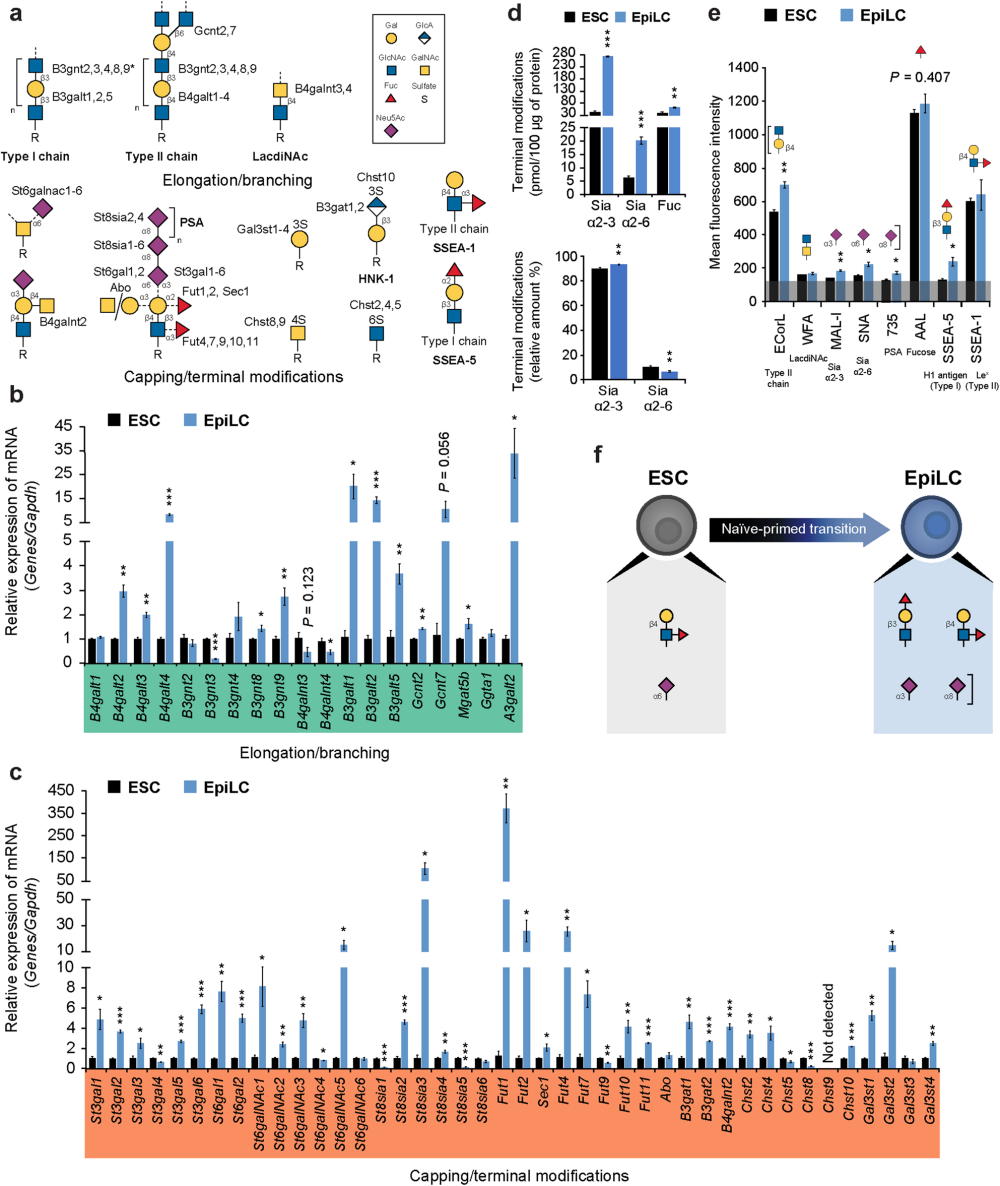
Pathway-non-specific structural and transcriptional analysis in ESCs and EpiLCs. (**a**) Schematic diagram of major pathway-non-specific structures. The asterisk denotes enzyme putative activity. (**b**) Real-time PCR analysis of elongation/branching enzymes normalized against *Gapdh* in ESCs and EpiLCs, and shown as a fold change relative to ESCs. (**c**) Real-time PCR analysis of capping/terminal modification enzymes normalized against *Gapdh* in ESCs and EpiLCs, and shown as a fold change relative to ESCs. (**d**) Total absolute amount of terminal modifications detected by MS in ESCs and EpiLCs (upper panel). Relative amount of total sialic acid in α2,3 and α2,6 configuration (lower panel). Fucose: Fuc; sialylation: Sia. (**e**) Pathway-non-specific structure profiling by FACS using specific lectins/Abs in ESCs and EpiLCs. Lectin/Ab specificities are stated below the labels and schematically represented above each histogram. The grey line at the bottom represents the negative control staining. (**f**) Model of pathway-non-specific alterations during ESC to EpiLC transition. Values are shown as means ± s.e.m. of three independent experiments in (**b**,**c**,**e**) and four independent experiments in (**d**). Significant values are indicated as *P < 0.05, **P < 0.01, and ***P < 0.001.

Glycosylation is a stepwise process involving more than 200 distinct glycosyltransferases and related enzymes in mammals. These can be classified as pathway-specific and pathway-non-specific, which generally include enzymes involved in biosynthetic steps overlapping different glycosylation classes, such as elongation/branching and capping/terminal modifications (Fig. 6a). Transcriptional analysis by Real-time PCR showed that *B3galt1,2,5* and *B4galt2-4,* involved in type I chain (Galβ1-3GlcNAc) and type II chain (Galβ1-4GlcNAc) structures formation, respectively, were enhanced, whereas LacdiNAc (GlcNAcβ1-4GalNAc)-specific enzymes *B4galnt3,4*, which we previously reported to positively regulate LIF signaling in ESCs^40^, were reduced in EpiLCs. Consistently with a higher level of KS (Fig. 4d), *B4galt4*, required for KS elongation^35^, was markedly increased. Moreover, upregulation of SSEA-3 at the structural level (Fig. 5d), correlated with a considerable higher expression of *B3galt5*, the enzyme involved in SSEA-3 synthesis on Gb^36^ (Fig. 6b). An overall upregulation of sialyltransferases, including *St8sia2,4,* the genes involved in polysialic acid (PSA) formation^41^, was observed in EpiLCs. Among them, *St6gal1,2*, the only sialyltransferases involved in *N*-glycans sialylation^41^, dramatically increased in EpiLCs, demonstrating a correlation between the observed enhancement of α2,6 sialic acid structures on *N*-glycans by MS (Fig. 2b) and the expression of relative enzymes. Consistently with the GSL shift from Gb to Gg series observed at the structural level (Fig. 5b,d), the expression of sialyltransferases involved in Gg extension, namely *St3gal2,5, St6galNAc3,5* and *St8sia3*, was substantially upregulated. Moreover, we detected a higher expression of *St3gal2*, the enzyme synthesizing SSEA-4 on Gb^36^, reflecting the increment observed by FACS (Fig. 5d). Fucosyltransferases expression strongly increased in EpiLCs, except for that of *Fut9*, the enzyme synthesizing SSEA-1^42^. Remarkably, *Fut1,2*, involved in the formation of SSEA-5, showed a striking upregulation. Furthermore, sulfotransferases mRNA levels were generally higher in EpiLCs. In particular, EpiLCs showed a considerable increase in *Chst2,4* (Fig. 6c)*,* the sole sulfotransferases that catalyze the formation of extended or branched capped mucin-type *O*-glycan structure^43^, confirming at the transcriptional level the data obtained by Meca-79 staining (Fig. 3d).

To obtain further insights into the major pathway-non-specific structures we used data obtained by MS and performed a FACS profiling using a combination of different lectins and Abs. Structures characterized by MS and FACS strongly correlated with the expression of the relative enzymes. We observed an increase in type II chain structures in EpiLCs, as indicated by *erythrina corallodendron* lectin (ECorL) staining, albeit Lewis x (Le^x^) type II structure (SSEA-1) was unchanged. SSEA-1 was previously shown to be synthesized by Fut4 and, with higher activity, by Fut9^44^, particularly, within the mouse embryo context^42^. Thus, SSEA-1 unaltered staining can be explained by decreased *Fut9* and a dramatic increase in *Fut4* expression. Conversely, H1 antigen, a type I structure known as SSEA-5, was substantially enhanced in EpiLCs and only detected at a negligible level in ESCs. This is consistent with the robust expression upregulation of the type I chain transferases *B3galt1,2,5* and the fucosyltransferases *Fut1,2,* demonstrating that type I structures increase during ESC to EpiLC transition. Overall fucosylation and sialylation levels were enhanced in EpiLCs, correlating with the observed increased expression of the fucose and sialic acid nucleotide transporters (Supplementary Fig. S10). Notably, sialic acid configuration shifted from an α2,6 to an α2,3 configuration in EpiLCs (Fig. 6d). Furthermore, PSA structure was specifically enhanced in EpiLCs, reflecting the upregulation of *St8sia2,4* expression (Fig. 6e,f and Supplementary Fig. S11).

### PRC2 contributes to glycosylation changes during ESC to EpiLC transition

**Figure 7:**
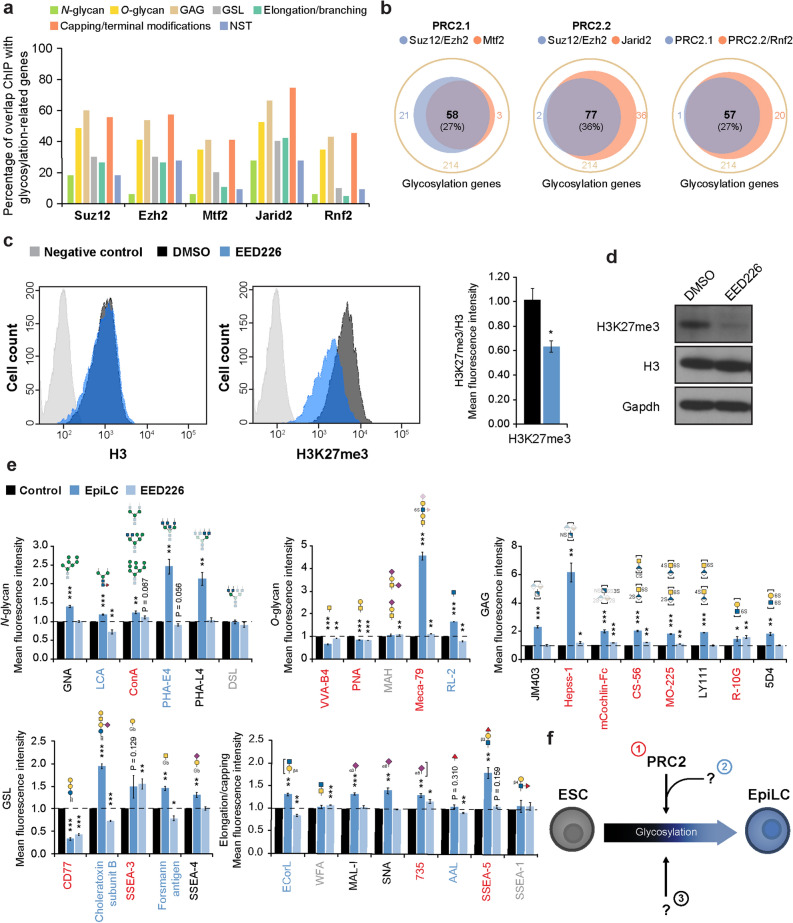
PRC2 contributes to glycosylation changes during ESC to EpiLC transition. (**a**) Analyzed ChIP-seq datasets were obtained from wild-type/untreated ESCs precipitated using: anti-Suz12 Ab (SRX1372665) (Ref.^46^), anti-Ezh2 Ab (SRX2528911) (Ref.^47^), anti-Mtf2 Ab (SRX2776968) (Ref.^48^), anti-Jarid2 Ab (SRX3738839) (Ref.^47^) and anti-Rnf2 Ab (SRX191072) (Ref.^49^). Percentages of ChIP occupancy per glycosylation class were determined within a range of ± 5 kb and with a threshold for statistical significance set as 50 (1 < 1E−05) calculated by peak-caller MACS2. (**b**) Venn diagrams showing the ChIP occupancies of PRC components overlap on glycosylation genes. (**c**) FACS analysis of H3 (left panel) and H3K27me3 (right panel) upon EED226 treatment for 48 h and relative histogram representing the fluorescence mean intensity shown as a fold change relative to that of DMSO-treated cells. Negative control: grey; DMSO: black; EED226: blue. (**d**) Representative cropped image of a western blot analysis of H3K27me3, H3 and Gapdh in ESCs treated with EED226 for 48 h. Full-length blots are presented in Supplementary Fig. S16. (**e**) Overall glycomic profiling by FACS using specific lectins/Abs in EpiLCs and EED226-treated ESCs, and shown as a fold change relative to ESCs and DMSO-treated ESCs (control), respectively. Lectin/Ab specificities are schematically represented above each histogram. The dotted line indicates the control fold change. (**f**) Schematic representation of the glycosylation regulatory network during ESC to EpiLC transition: PRC2 direct regulation (1, red), PRC2 regulation together with other unidentified factor(s) (2, blue), and PRC2-independent pathway(s) (3, black) (the grey label indicates unchanged structures). Values are shown as means ± s.e.m. of three independent experiments. Significant values are indicated as *P < 0.05, **P < 0.01, and ***P < 0.001.

Since the glycosylation profile dynamically changes during ESC to EpiLC transition, we hypothesized the presence of a defined regulatory network. To identify putative candidates, we performed an in-depth analysis of previously published chromatin immunoprecipitation sequencing (ChIP-seq) datasets obtained in ESCs using the ChIP-Atlas comprehensive database^45^ (https://chip-atlas.org), searching for factors that are enriched at the glycosyltransferase promoter regions. As a result, we observed that PRC2 components occupy a great variety of glycosylation-related genes across all glycosylation classes in ESCs (Fig. 7a and Supplementary Fig. S12). PRC2, whose core components are Suz12, Eed, and either Ezh2 or Ezh1, is a chromatin-remodeling complex which catalyzes the H3K27me3 modification to promote gene repression. PRC2 can associate with other factors that regulate its chromatin recruitment, such as Mtf2 or Jarid2, to form two different subtypes of PRC2 named PRC2.1 and PRC2.2, respectively. In addition, PRC2 can synergically repress gene expression together with PRC1, which is composed by core components, such as Rnf2^23,24^. Further analysis of previously published ChIP-seq data indicated that PRC2.1, PRC2.2 and Rnf2 (PRC1) act cooperatively to regulate around 27% of the glycosylation-related genes in ESCs (Fig. 7b). Moreover, a global alteration in the epigenetic state of glycosylation-related genes was observed in EpiLCs compared to ESCs with changes in PRC2-related histone modification H3K27me3 and its counterpart H3K27ac, which promote gene silencing and activation, respectively; promoter activation histone marker H3K4me3; and RNA polymerase II binding^23^ (Supplementary Fig. S13 and Supplementary Table S1). Together, these data suggest that PRC2 is involved in glycosylation changes occurring during the transition from ESCs to EpiLCs.

To test this hypothesis, we treated ESCs with the PRC2 inhibitor EED226^50^ for 48 h and examined the effects on the glycome. EED226 treatment resulted in a considerable reduction of the H3K27me3 modification (Fig. 7c,d). Glycomic profiling was performed by FACS analysis. As a result, we observed significant alterations in a wide range of structures, confirming that PRC2 is involved in glycosylation regulation in ESCs (Supplementary Fig. S14a). Next, we compared the glycosylation alterations observed by FACS during the ESC to EpiLC transition and in ESCs after EED226 treatment. Strikingly, a large number of structures were increased or decreased both in EpiLCs and EED226-treated ESCs, indicating a direct modulation by PRC2 (red in Fig. 7e). Conversely, other structures showed an opposite trend in EpiLCs and EED226-treated ESCs, suggesting the presence of other regulatory component(s) (blue in Fig. 7e). Finally, some structures were altered in EpiLCs but unchanged in EED226-treated ESCs, implying the presence of PRC2-independent pathway(s) (black in Fig. 7e). Transcriptional analysis of PRC2 core components by RNA-seq in ESCs and EpiLCs showed a decrease at the transcriptional level of the PRC2 core component *Eed* (Supplementary Fig. S14b). In addition, global H3K27me3 has been previously reported to be drastically reduced and redistributed upon ESC differentiation to EpiLCs^51^, providing further evidence supporting our PRC2-mediated glycosylation regulatory network model during ESC to EpiLC transition. To further confirm that PRC2 is involved in glycan changes during the ESC to EpiLC transition we examined the glycosyltransferase and related enzyme expression difference in ESCs vs EpiLCs and EED226 untreated vs EED226 treated samples by RNA-seq. Similar to the changes observed at the structural level, we observed three different patterns of expression: expression increased or decreased both in EpiLCs and EED226-treated ESCs (43% of the glycosyltransferases and related genes); or following an opposite trends of expression (22% of the glycosyltransferases and related genes); or unaffected by PRC2 treatment and changed during the naïve-to-primed transition (29% of the glycosyltransferases and related genes) (Supplementary Fig. S14c and Supplementary Table S5). Collectively, these data led us to postulate the presence of at least three coordinated pathways that control glycosylation dynamics during EpiLC differentiation (Fig. 7f).

In conclusion, our findings demonstrate for the first time the presence of a developmentally regulated network orchestrating overall glycosylation changes and identified PRC2 as a key component involved in this process.

## Discussion

**Figure 8:**
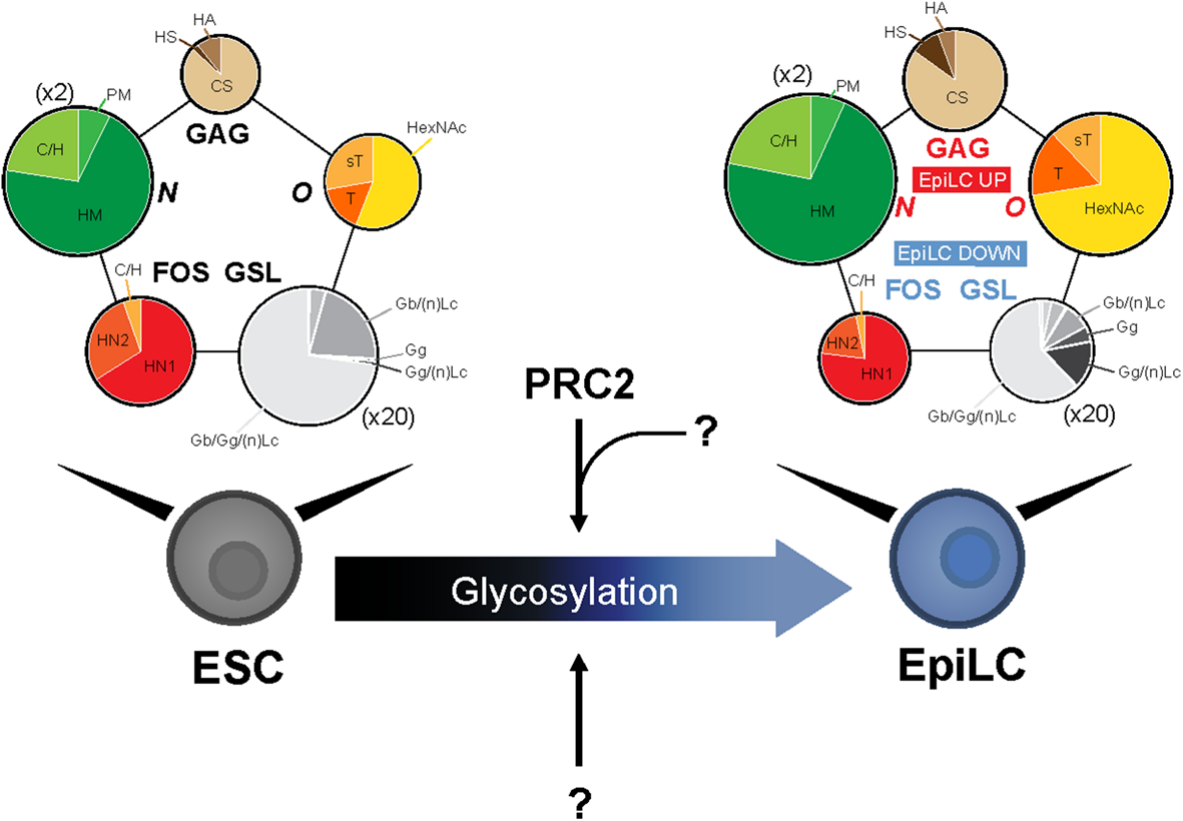
Schematic model of glycosylation dynamics and regulatory network during the ESC (naïve) to EpiLC (primed) transition. The overall glycosylation composition changes dramatically during the naïve to primed transition. PRC2 is a key component of the developmentally regulated network orchestrating glycosylation dynamics. The size of each pie chart reflects the absolute mean quantity of glycans (pmol/100 μg of protein). *N*-linked and GSL pie charts size is scaled 2 and 20 times, respectively.

The pivotal role of glycosylation during development and in determining stem cell identity across different species is becoming increasingly clear^11^. Previous reports characterized the glycomic profiles of mouse ESCs, conventional human ESCs (hESCs) and human induced pluripotent stem cells (hiPSCs), which are in a primed state, tumors and late differentiating cells^20–22^. In the present study, we performed a comprehensive and comparative analysis to investigate the glycosylation dynamics during the pluripotency state transition from ESCs to EpiLCs, which have been recently suggested to be in an intermediate developmental stage between the naïve state and the primed state, named formative state^52^. As a result, we demonstrated that glycosylation undergoes dramatic alterations from early stages of development, and we identified PRC2 as a key component of the network orchestrating these changes (Fig. 8).

Pluripotent stem cells (PSCs) in the primed state exhibit significant differences compared to naïve ESCs, such as a flat morphology, glycolytic metabolism, slow proliferation, and closer chromatin^53^*.* During the naïve-to-primed transition, RAS activation mediates the epithelial-to-mesenchymal transition (EMT), characterized by the switch from epithelial cadherin (Ecad) to neural cadherin (Ncad)^54^; a process similar to cancer progression and tightly regulated by glycosylation^55,56^. Indeed, *N*-glycosylation function in the EMT during tumorigenesis has been widely reported: 1–6 branching structure on Ecad promotes its clearance from the cell surface and inhibits Ncad-mediated cell–cell adhesion. Furthermore, core fucosylation weakens Ecad cell–cell adhesion in lung cancer^55^. In addition, an increased level of sialylated glycans^56^ and reduced GSL^57^ were documented to correlate with EMT progression. Similarly, we detected enhanced 2,6 branching, core fucosylation (Fig. 2b,e), and total sialylated glycans (Fig. 6d), followed by a sharp reduction in GSL (Fig. 5b), suggesting a shared EMT regulation by glycosylation across different biological contexts. RAS activation was also reported to be linked to a metabolic shift from oxidative phosphorylation to glycolysis, and subsequent closer chromatin during the naïve-to-primed differentiation^54^. Importantly, RAS is downstream of FGF signaling, which requires *N*-sulfation of HS. Indeed, *Ndst1/2*^*−/−*^ ESCs are unable to exit from the naïve pluripotent state^11^. Moreover, Myc amount, which promotes the ESCs’ proliferative program and thus the proliferation speed, is inversely correlated with FGF-ERK activation^58^, underlining the functional importance of FGF signaling regulation by HS. Accordingly, we observed a dramatic increase in *Ndst2-4* expression and *N*-sulfated HS staining (Fig. 4c,d). In the light of previous reports, our data emphasize that glycosylation dynamic changes contribute and partially drive the major phenotypical alterations occurring during the naïve-to-primed transition, thus underlining the importance of mechanistically dissecting the role of glycosylation during developmental transitions in vitro and in vivo.

Comparative analysis of total glycomes allows the identification of pluripotency biomarker candidates^21^. Indeed, our data confirmed previously established pluripotency markers, such as SSEA-1,3,4 and Forsmann antigen, which are expressed at a similar levels in ESCs and EpiLCs. Moreover, we demonstrated that SSEA-5 is specifically expressed in EpiLCs, augmenting previous studies performed in conventional hESCs and hiPSCs^59^. In addition, we identified a wide range of novel structures across all glycosylation classes that are specifically enhanced in EpiLCs but not detected or detected at very low levels in ESCs providing additional markers to distinguish between the naïve and primed pluripotent states: bisecting GlcNAc and 2,6 branched tri-/tetra-antennary complex *N*-glycans, extended or branched capped mucin-type *O*-glycan structure, *N*-unsubstituted GlcN, *N*-sulfated GlcN, and 3-*O*-sulfated HS, CS-A, C, D and E units, and PSA.

Expanded potential stem cells (EPSCs) can contribute to extraembryonic tissues in intraspecies chimeras and hence are totipotent stem cells^60^. Moreover, Dux overexpression converts ESCs into 2-cell-embryo-like (2C-like) cells^61^. The recent establishment of culture conditions to reprogram mouse ESCs into EPSCs or 2C-like cells allowed the in vitro investigation of the totipotent state. Despite the most suitable in vitro system to model the totipotent state is still under debate^62^, these models allowed the identification of some molecular features of totipotent cells^60–62^, providing an invaluable tool to characterize the totipotent state. Glycosyltransferases expression dramatically diverges between EPSCs/2C-like cells and ESCs in vitro^60,61^, and between the embryo cleavage stage and the ICM in vivo^61^. Thus, it will be of great interest to examine the glycosylation dynamics during the reprogramming process from ESCs to EPSCs and 2C-like cells to establish novel totipotency biomarkers and obtain mechanistic insights into the transition from the totipotent to the pluripotent state.

PRC2 regulates early embryonic specification processes by repressing crucial developmental genes^63^. Indeed, deficiency in PRC2 core components *Eed*, *Suz12*, or *Ezh2* results in embryo lethality around E7.5-E8.5 due to gastrulation defects^24^. Moreover, PRC2 was previously reported to be essential to maintain the primed but not the naïve state of pluripotency both in mouse and human^64^. Here, we showed for the first time that PRC2 contributes to overall glycosylation alterations occurring during the ESC to EpiLC transition. Recently, ISY1 has been reported to modulate the biogenesis of a large subset of crucial miRNAs during the transition from ESCs to EpiLCs^27^. Moreover, PRC2 represses around 512 developmental regulators in ESCs^63^. Thus, the glycosylation changes we observed in EpiLCs are likely to be the result of the synergic action of PRC2 on glycosylation-related genes expression together with other component(s) involved both directly and indirectly in the glycosylation pathway and PRC2-independent pathway(s). In addition, PRC1 and PRC2 activity is directly modulated by *O*-GlcNAc^65^, suggesting a metabolically regulated network controlling the glycomic profile. PRDM14 is a critical pluripotency determinant conserved among mice and humans that suppresses developmental genes in ESCs by binding and recruiting PRC2 to the target genes^66,67^. Given this common pluripotency safeguard mechanism, it will be particularly interesting to investigate PRDM14 role in the PRC2-mediated glycosylation network, and whether the observed effects are translatable to human PSCs or are species-specific.

Glycosylation is a developmentally coordinated post-translational modification^10,11^. Previous studies have identified glycosylation class-specific key regulators. For instance, hepatocyte nuclear factor 1α (HNF1α) was demonstrated to control *N*-glycan fucosylation of human plasma proteins^68^. More recently, autism susceptibility candidate 2 (Auts2) was shown to drive GSL metabolic switch during neural differentiation from mouse ESCs^37^. Nonetheless, regulation of overall glycosylation dynamics has remained unknown. Our study identified PRC2 as a key factor involved in the glycosylation changes occurring during naïve to primed transition. Our findings are the first demonstration that glycome complex alterations occurring during developmental transitions are orchestrated by a defined regulatory network. Consequently, it will be important to characterize the glycomic dynamics in a variety of developmental stages and cell types in order to identify transition-specific glycosylation regulatory components. Glycosylation is involved in a broad range of cellular processes^9^. Not surprisingly, aberrant forms of glycosylation are observed in all types of cancer^56^. Thus, we postulate the existence of glycosylation regulatory networks acting during tumorigenic processes, which identification will allow the development of novel therapeutic approaches. In conclusion, our findings provide a solid groundwork for further investigations in basic research and translational medicine.

## Methods

### Cell culture

R1 ESC line^69^ was maintained on mouse embryonic fibroblasts (MEFs) that were prepared from embryos at embryonic day 14.5 and inactivated with 10 μg mitomycin C (Sigma-Aldrich). ESCs were maintained in ESC medium consisting of DMEM (Gibco) supplemented with 15% fetal bovine serum (FBS) (Nichirei Biosciences), 1% penicillin/streptomycin (Gibco), 0.1 mM 2-mercaptoethanol (Gibco), 0.1 mM non-essential amino acids (Gibco) and 1000 U/mL LIF (Chemicon International). All animal experiments were approved by the Animal Care and Use Committee for Soka University and were performed in accordance with relevant guidelines and regulations for animal care.

To induce EpiLC differentiation, ESCs maintained on MEF feeder cells were passaged on gelatin-coated 60-mm dishes, and cultured for 30 min in ESC medium containing LIF to completely remove MEF feeder cells 24 h prior induction. Subsequently, supernatants containing ESCs were seeded at low density in gelatin-coated 60-mm dishes containing ESC medium in the presence of 1 μM PD0325901 (Wako), 3 μM CHIR99021 (Wako) and LIF. The following day, EpiLC induction was performed; ESC samples were collected for further analysis or seeded at 1 × 10^6^ in gelatin-coated 60-mm dishes containing ESC medium without LIF for 24 h. Subsequently, EpiLC medium, consisting of DMEM/F12 (Gibco) supplemented with 20% knockout serum replacement (KSR) (Gibco), 2 mM L-glutamine (Invitrogen), 1% penicillin/streptomycin (Gibco), 0.1 mM 2-mercaptoethanol (Gibco), 0.1 mM nonessential amino acids (Gibco), 30 ng/ml FGF2 (Wako) and 0.6 μM JAK inhibitor (JAKi) (Santa Cruz Biotechnology), was added. EpiLC medium was changed daily and EpiLCs were collected for further analysis 72 h post-induction.

To analyze the effect of PRC2 inhibition, ESCs were cultured in ESC medium containing 10 μM EED226 in the presence of LIF for 48 h.

### Real-time PCR and RNA-sequencing

Total RNA was extracted from cells using TRIzol reagent (Invitrogen) and reverse-transcribed using a Superscript II First Strand Synthesis Kit (Invitrogen) and oligo-dT primers. Real-time PCR was performed with an ABI PRISM 7700 Sequence Detection System (Applied Biosystems) and SYBR Green Master Mix (Roche). The relative amount of each mRNA was normalized against the amount of *Gapdh* mRNA in the same sample. The primer sets used are listed in Supplementary Table S2.

The RNA-seq libraries were prepared from total RNA using the TruSeq Stranded mRNA Prep kit (Illumina) according to the manufacturer’s instructions. 100 bp single-read sequencing was performed by NovaSeq6000 (Illumina).

### Immunostaining

Cells were fixed with 4% paraformaldehyde in PBS and washed with PBS. Fixed cells were blocked with 1% BSA/0.3% Triton X-100 in PBS. For primary labeling, cells were incubated with anti-Oct3/4 Ab (Santa Cruz Biotechnology; sc-5279; 1:100), anti-Nanog Ab (ReproCELL; RCAB001P; 1:100) or anti-Otx2 Ab (Millipore; AB9566; 1:100). Later, the cells were stained with anti-mouse IgG Alexa Fluor 488-conjugated (Life Technologies; A-11001; 1:300) or anti-rabbit IgG Alexa Fluor 488-conjugated (Life Technologies; A-11008; 1:300) and Hoechst 33342 (Invitrogen; H3570; 5 μg/mL). Images were obtained using an LSM 700 confocal laser microscope (Carl Zeiss).

### Western blotting

Cells were lysed with lysis buffer (50 mM Tris-HCl [pH 7.4], 150 mM NaCl, 1% Triton X-100, 5 mM EDTA, 1 mM Na_3_VO_4_, 10 mM NaF and protease inhibitors). Protein samples were separated on an SDS polyacrylamide gel and transferred onto polyvinylidene fluoride membranes (Millipore). Membranes were blocked using 1% or 5% BSA and incubated with the following primary antibodies: anti-p-ERK1/2 Ab (Cell Signaling; #9101L; 1:1000), anti-ERK1/2 Ab (Cell Signaling; #9102L; 1:1000), anti-Oct3/4 Ab (Santa Cruz Biotechnology; sc-5279; 1:1000), anti-Nanog Ab (ReproCELL; RCAB001P; 1:1000), anti-Otx2 Ab (Millipore; AB9566; 1:1000), anti-H3 Ab (Cell Signaling; #9715; 1:1000), anti-H3K27me3 Ab (Cell Signaling; #9733; 1:1000) or anti-Gapdh Ab (Santa Cruz Biotechnology; sc-32233; 1:1000). Membranes were then incubated with anti-rabbit IgG (Cell Signaling; #7074; 1:10,000) or anti-mouse IgG (Cell Signaling; #7076; 1:10,000) HRP-conjugated secondary Abs. Membranes were then washed and developed with ECL Plus reagents (GE Healthcare).

### Lectin purification and biotinylation

*Maackia amurensis* hemagglutinin (MAH) and leukoagglutinin (MAL), *Erythrina corallodendron* lectin (ECorL), *Wisteria floribunda* agglutinin (WFA) and *Vicia villosa* agglutinin-B4 (VVA-B4) were purified from seeds (F.W. Schumacher Co., Sandwich, MA, USA) as previously described^70–73^. Biotinylation of purified lectins was performed as follows: a few mg of each purified lectin was solubilized with 50 mM NaHCO_3_. Solubilized lectins were incubated with 50 μg of sulfo-NHS-biotin (Thermo Fisher Scientific) in H_2_O at 4 °C for 16 h. Later, 50 μL of 1 M Tris buffer (pH 8.0) were added. After 2 h, the reaction mixture was applied onto a PD-10 desalting column (GE Healthcare), and eluted with 50 mM phosphate buffer (pH 7.8). Fractions containing biotinylated lectins were selected by monitoring absorbance at 280 nm.

### Construction of plasmid for mouse cochlin-Fc fusion protein and its expression in HEK293 cells

The cDNA encoding mouse cochlin (Genbank: NM_00729.5), which specifically recognizes 3-*O*-sulfation on HS (unpublished data), was amplified by PCR from mouse spleen cDNA, using the following primers: 5′-GTTCTCTGTGTTTGGGAACAT-3′ and 5′-TCCTCAAGAGAGCAGCCTCC-3′. To express mouse cochlin fused to human IgG-Fc (mCochlin-Fc), mouse cochlin cDNA was amplified by PCR and inserted between the EcoRI and XhoI sites of a pCAGGS-Fc vector. HEK293 cells were transfected with the purified plasmid using Lipofectamine 2000 and cultured in D10 medium containing 2 μg/mL puromycin for a week. Cultured media was collected, and mCochlin-Fc was purified using a column of Protein A-Sepharose column (GE healthcare).

### FACS analysis

A single cell suspension for cell surface molecules staining was obtained using 0.02% EDTA/PBS. Subsequently, 2–3 × 10^5^ cells were collected and washed in FACS buffer (0.5% BSA (Iwai), 0.1% sodium azide (Sigma-Aldrich) in PBS). After washing, the cell suspension was incubated with Abs or lectins in FACS buffer. For internal molecules analysis, cells were harvested with 0.25% trypsin/EDTA (Thermo Fisher Scientific) and fixed/permeabilized for 30 min with 100% methanol (Wako) before staining. Samples were then analyzed using a BD FACSAria III Cell Sorter (Becton Dickinson). Cells were gated to exclude debris, dead cells (identified by propidium iodide staining; Sigma-Aldrich), and doublets. The primary and secondary Abs and lectins used are listed in Supplementary Table S3.

### Sample preparation for glycome analysis of various glycoconjugates

Cultured ESCs and EpiLCs (> 1 × 10^6^ cells) were washed 5 times with PBS and collected using a scraper. Collected cells were resuspended in 100 mL of PBS and homogenized using an Ultrasonic Homogenizer (TAITEC, Saitama, Japan). Cell lysates were precipitated with EtOH and subsequently the proteinous pellet and supernatant fractions were separated by centrifugation^22,74,75^. The resulting pellet was dissolved in H_2_O and cellular protein concentration was measured using a BCA protein assay kit (Thermo Fisher Scientific). The pellet fractions corresponding to 25 μg, 50 μg, and 100 μg of proteins were used for *N*-glycans, *O*-glycans, and GAG analyses, respectively. The supernatant fraction corresponding to 100 μg of proteins was concentrated for GSL and FOS analyses. Glycomic analyses of *N*-glycans, GSL, and FOS were performed by glycoblotting methods combined with the SALSA procedure^76,77^. *O*-glycome analysis was performed by β-elimination in the presence of pyrazolone analogues (BEP) method, and GAG were measured by HPLC as previously described^21^. This methodology allows a comparative analysis of glycomes. The deduced composition and absolute amount of detected structures is listed in Supplementary Table S4.

### Statistical analysis

An unpaired two-tailed Student's *t*-test was used to calculate *P* values. Statistical significance is denoted by asterisks: *P < 0.05; **P < 0.01; and ***P < 0.001. Error bars represent s.e.m.

## Supplementary Information

Supplementary Information.

Supplementary Table S1.

Supplementary Table S2.

Supplementary Table S3.

Supplementary Table S4.

Supplementary Table S5.

## Data Availability

The ChIP-seq datasets analyzed in this study are publicly available at NCBI Sequence Read Archive [Accession Numbers: SRX4488301, SRX4488308, SRX4488293, SRX4488300, SRX4488285, SRX4488292, SRX4488317 and SRX4488324 (Ref.^7^); SRX1372665 (Ref.^46^); SRX2528911 and SRX3738839 (Ref.^47^); SRX2776968 (Ref.^48^); SRX191072 (Ref.^49^)]. RNA-seq data generated for this study has been deposited in the GEO repository under accession number GSE161278. The data that support the findings of this study are available from the corresponding author upon reasonable request.
