# Supplementary material for: A defined glycosylation regulatory network modulates total glycome dynamics during pluripotency state transition
Source: Sci Rep. 2021 Jan 14;11:1276. doi: 10.1038/s41598-020-79666-4 (PMC7809059; doi:10.1038/s41598-020-79666-4)
Supplement: Supplementary file 1 — Supplementary Information. [file 41598_2020_79666_MOESM1_ESM.pdf]

A defined glycosylation regulatory network modulates total glycome dynamics during pluripotency state transition

Federico Pecori[1], Ikuko Yokota[2], Hisatoshi Hanamatsu[2], Taichi Miura[1,7], Chika Ogura[1], Hayato Ota[1], Jun-ichi Furukawa[2], Shinya Oki[3], Kazuo Yamamoto[4], Osamu Yoshie[5] & Shoko Nishihara*[1,6].

[1] Laboratory of Cell Biology, Department of Bioinformatics, Graduate School of Engineering, Soka University, 1-236 Tangi-machi, Hachioji, Tokyo 192-8577, Japan

[2] Department of Advanced clinical glycobiology, Faculty of Medicine and Graduate School of Medicine, Hokkaido University, Kita 15, Nishi 7, Kita-ku, Sapporo, Hokkaido 060-8638, Japan

[3] Department of Drug Discovery Medicine, Graduate School of Medicine, Kyoto University, 53 Shogoin Kawahara-cho, Sakyo-ku, Kyoto 606-8507, Japan

[4] Department of Integrated Biosciences, Graduate School of Frontier Sciences, The University of Tokyo, 5-1-5 Kashiwanoha, Kashiwa, Chiba 277-8562, Japan

[5] Health and Kampo Institute, 1-11-10 Murasakiyama, Izumi, Sendai, Miyagi 981-3205, Japan

[6] Glycan & Life System Integration Center (GaLSIC), Faculty of Science and Engineering, Soka University, 1-236 Tangi-machi, Hachioji, Tokyo 192-8577, Japan

[7] Present address: National Institute of Radiological Sciences (NIRS), National Institutes for Quantum and Radiological Science and Technology, 4-9-1 Anagawa, Inage-ku, Chiba 263-8555, Japan

Correspondence and requests for materials should be addressed to S.N. (email: shoko@soka.ac.jp)

Supplementary Figures

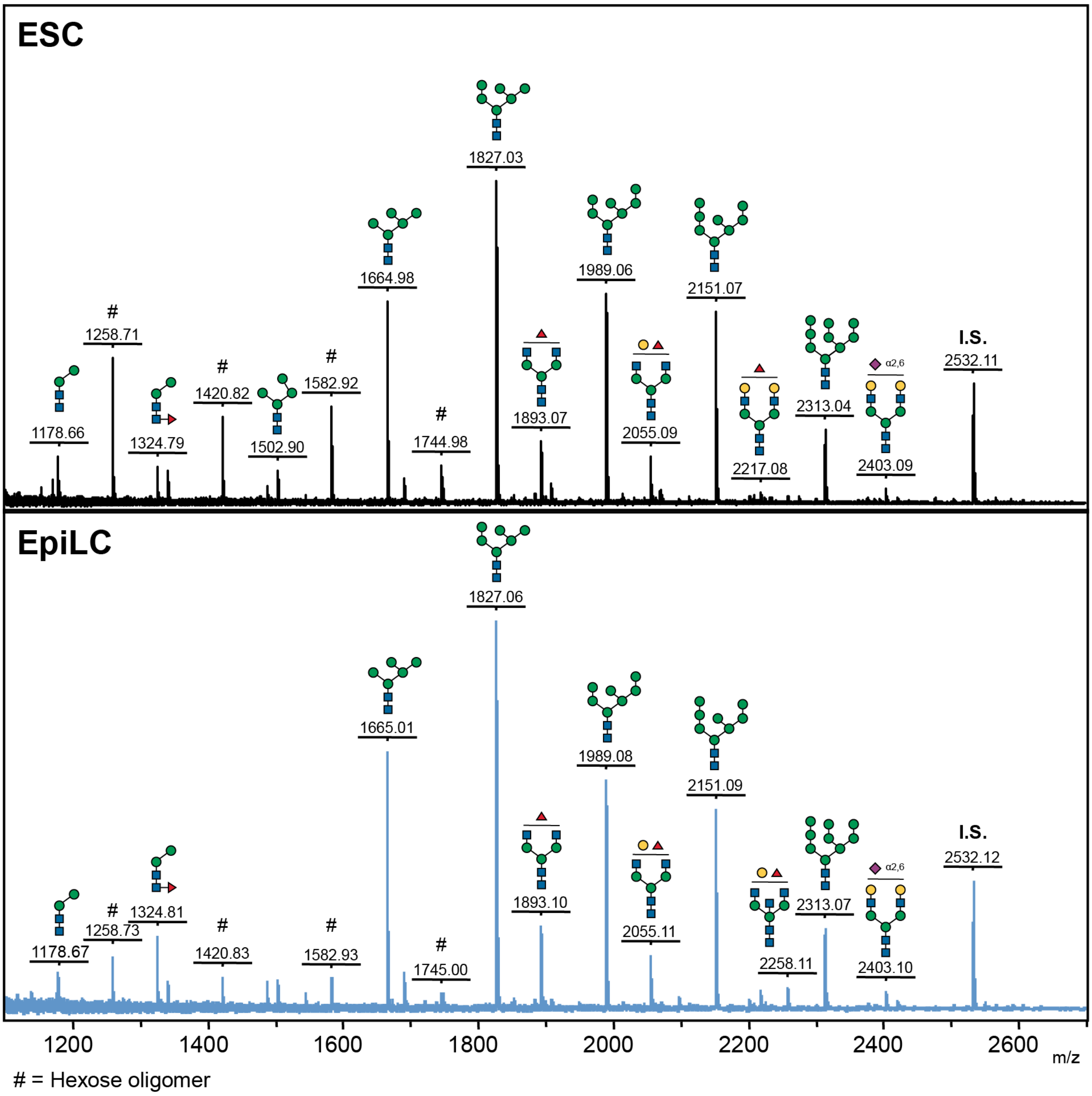

Supplementary Fig. S1 *N*-glycome quantification. Representative *N*-glycome MS spectra of ESCs (upper panel) and EpiLCs (lower panel). To allow intensity comparison across ESCs and EpiLCs, MS signals in ESC and EpiLC spectra are scaled to the same I.S. (internal standard) signal area. Representative estimated structures are shown. The absolute amount of detected structures is listed in Supplementary Table S4.

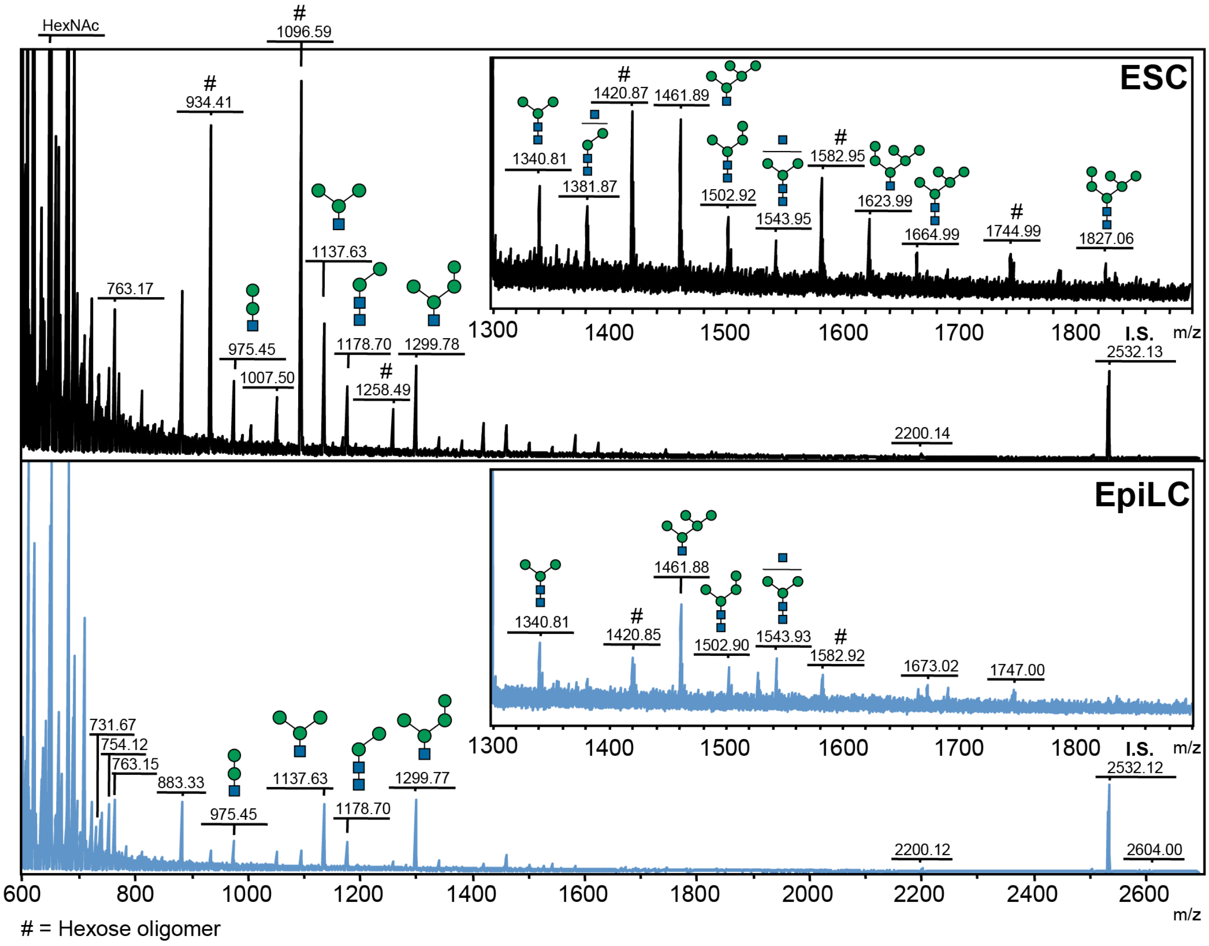

Supplementary Fig. S2 FOS quantification. Representative FOS MS spectra of ESCs (upper panel) and EpiLCs (lower panel). To allow intensity comparison across ESCs and EpiLCs, MS signals in ESC and EpiLC spectra are scaled to the same I.S. (internal standard) signal area. The absolute amount of detected structures is listed in Supplementary Table S4.

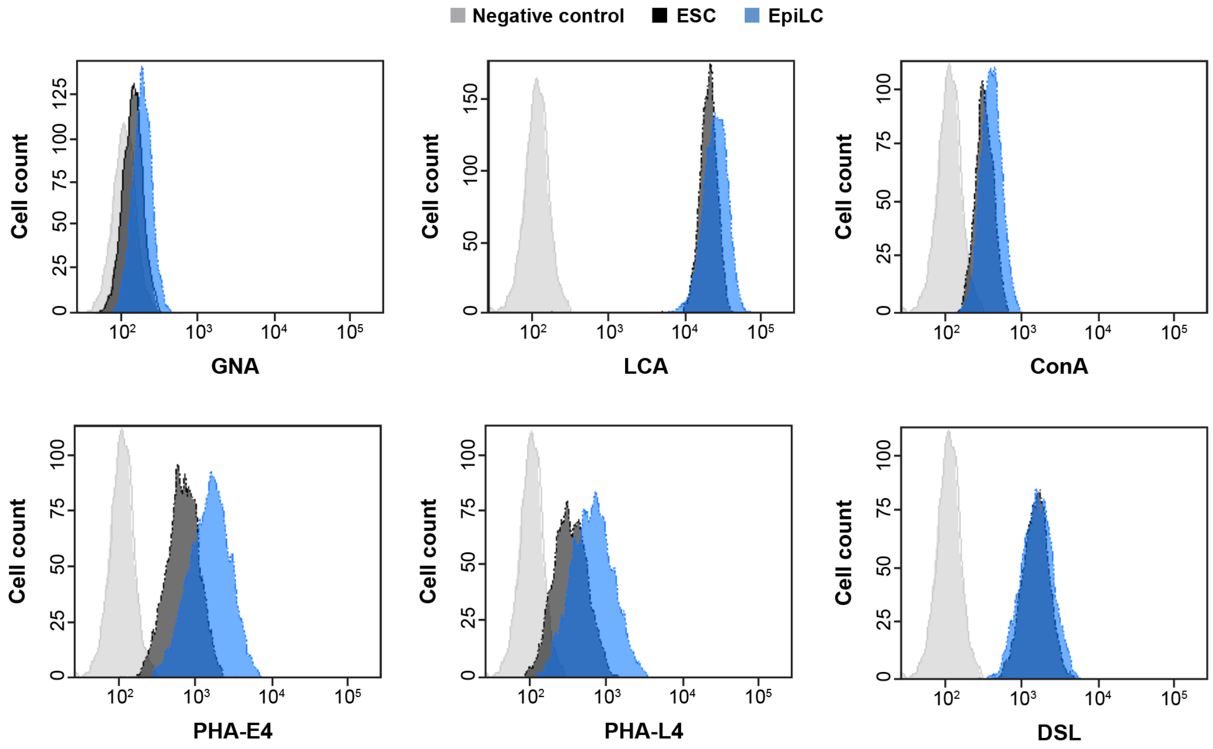

Supplementary Fig. S3 *N*-glycan structure profiling. Representative FACS histograms of ESCs and EpiLCs. Negative control: grey; ESC: black; EpiLC: blue.

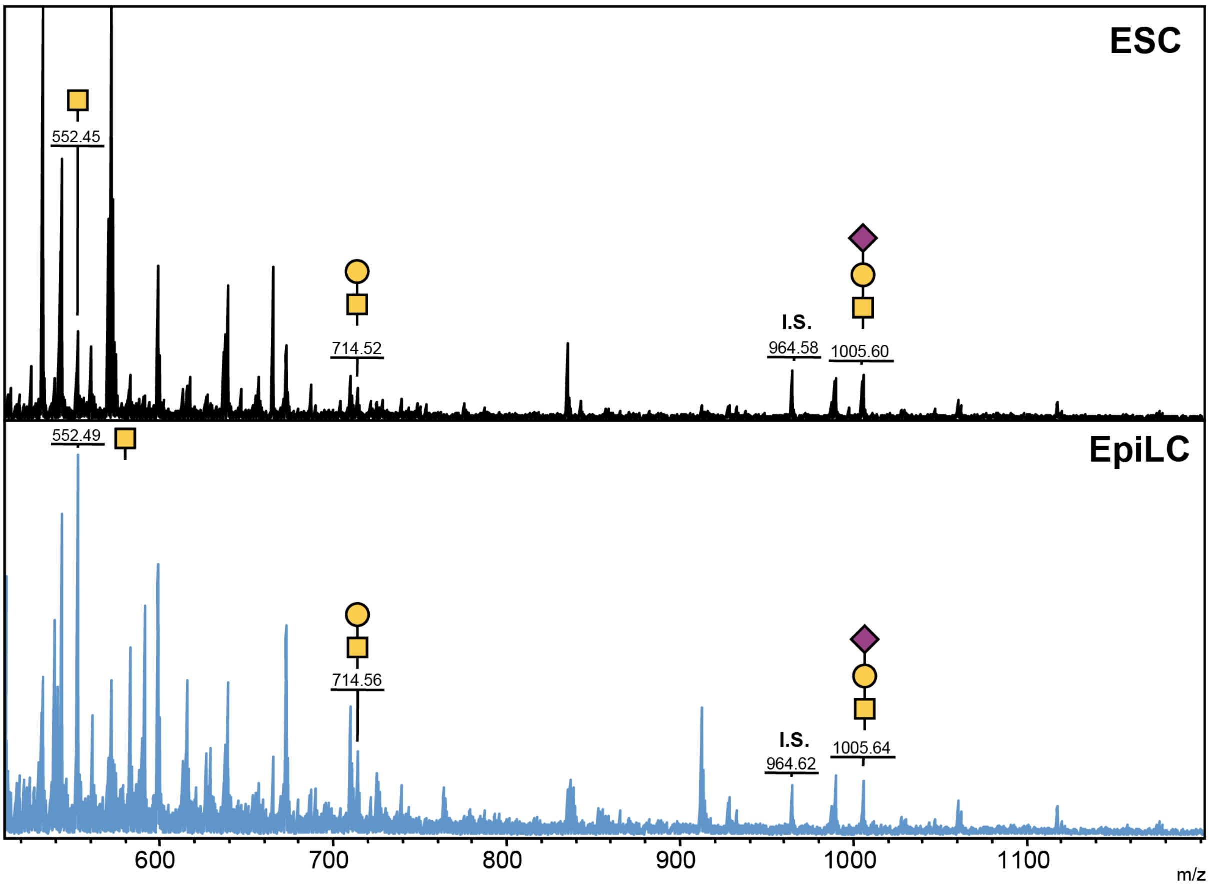

Supplementary Fig. S4 *O*-glycome quantification. Representative *O*-glycome MS spectra of ESCs (upper panel) and EpiLCs (lower panel). To allow intensity comparison across ESCs and EpiLCs, MS signals in ESC and EpiLC spectra are scaled to the same I.S. (internal standard) signal area. Estimated structures are shown. The absolute amount of detected structures is listed in Supplementary Table S4.

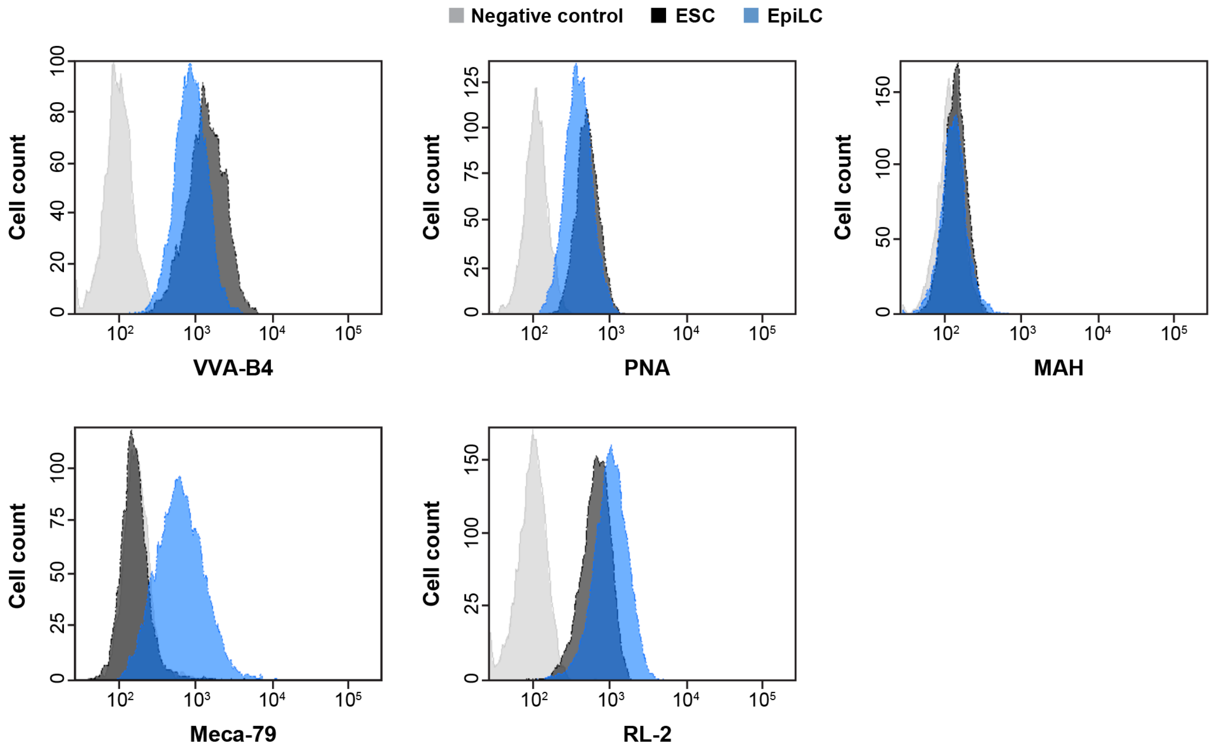

Supplementary Fig. S5 *O*-glycan structure profiling. Representative FACS histograms of ESCs and EpiLCs. Negative control: grey; ESC: black; EpiLC: blue.

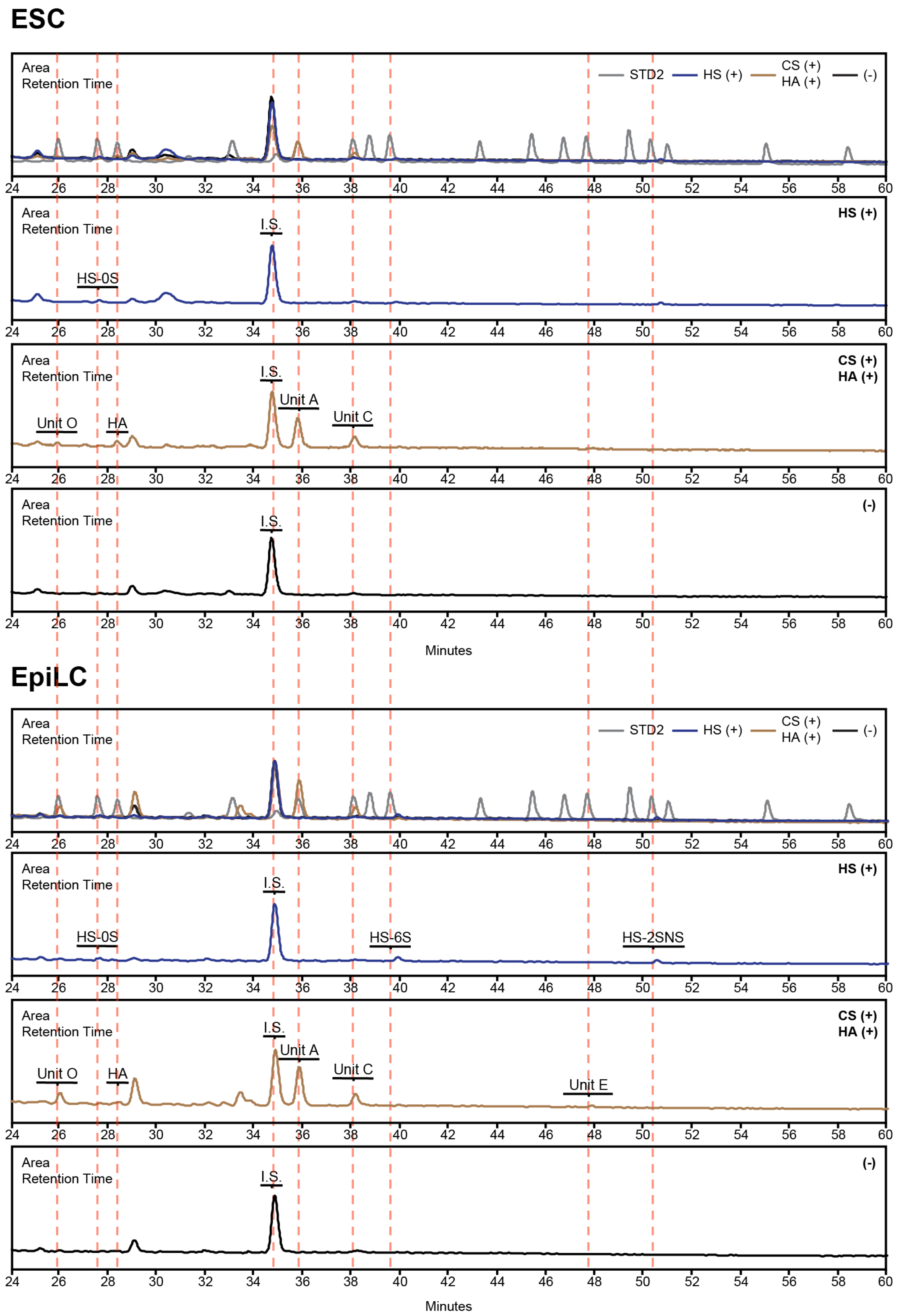

Supplementary Fig. S6 GAG structure quantification. Representative GAG HPLC chromatogram of ESCs (upper panel) and EpiLCs (lower panel). To allow intensity comparison across ESCs and EpiLCs, HPLC signals are scaled to the same I.S. (internal standard) signal area. Estimated structures are labeled on the

chromatogram. The absolute amount of detected structures is listed in Supplementary Table S4.

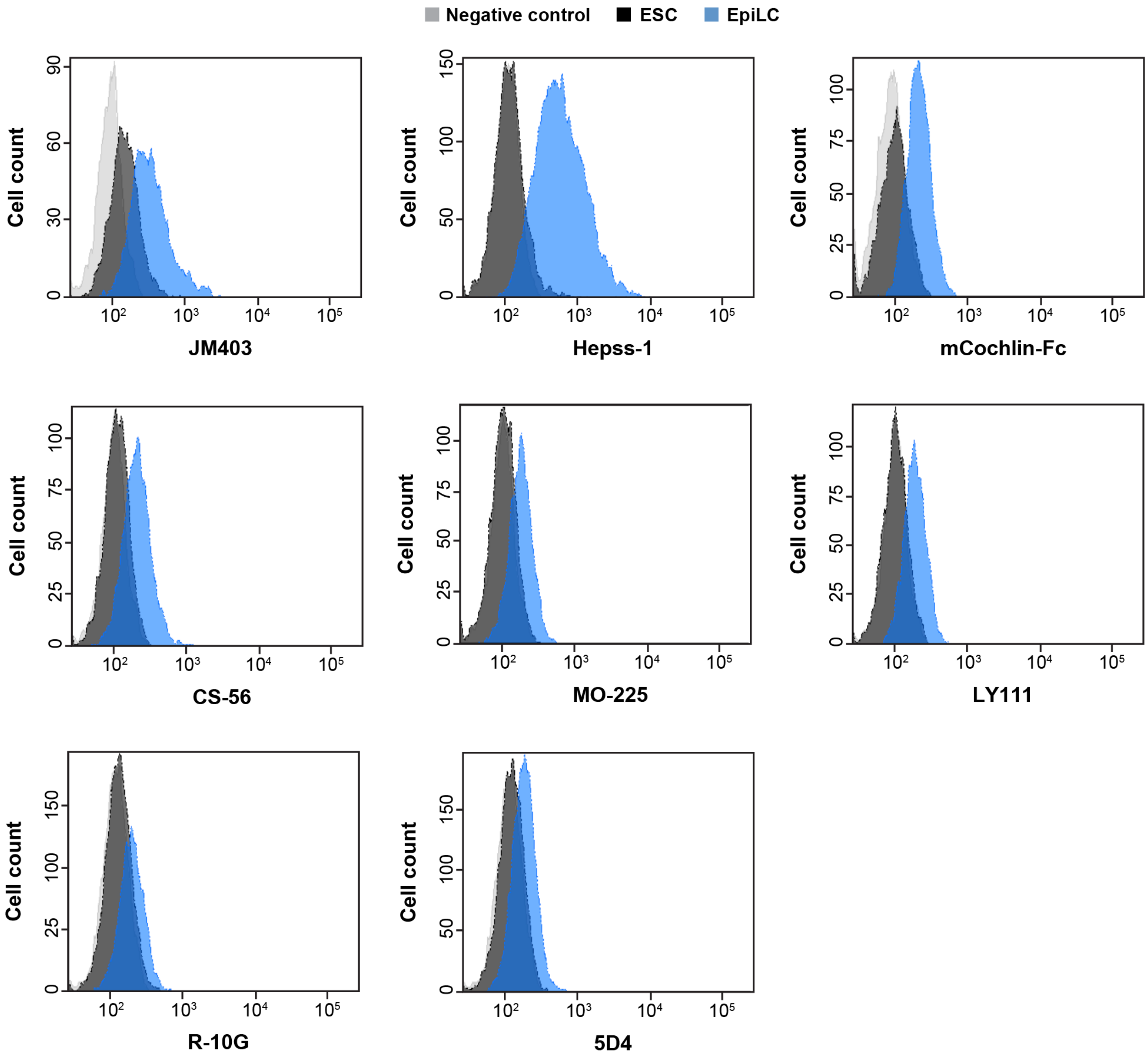

Supplementary Fig. S7 GAG structure profiling. Representative FACS histograms of ESCs and EpiLCs. Negative control: grey; ESC: black; EpiLC: blue.

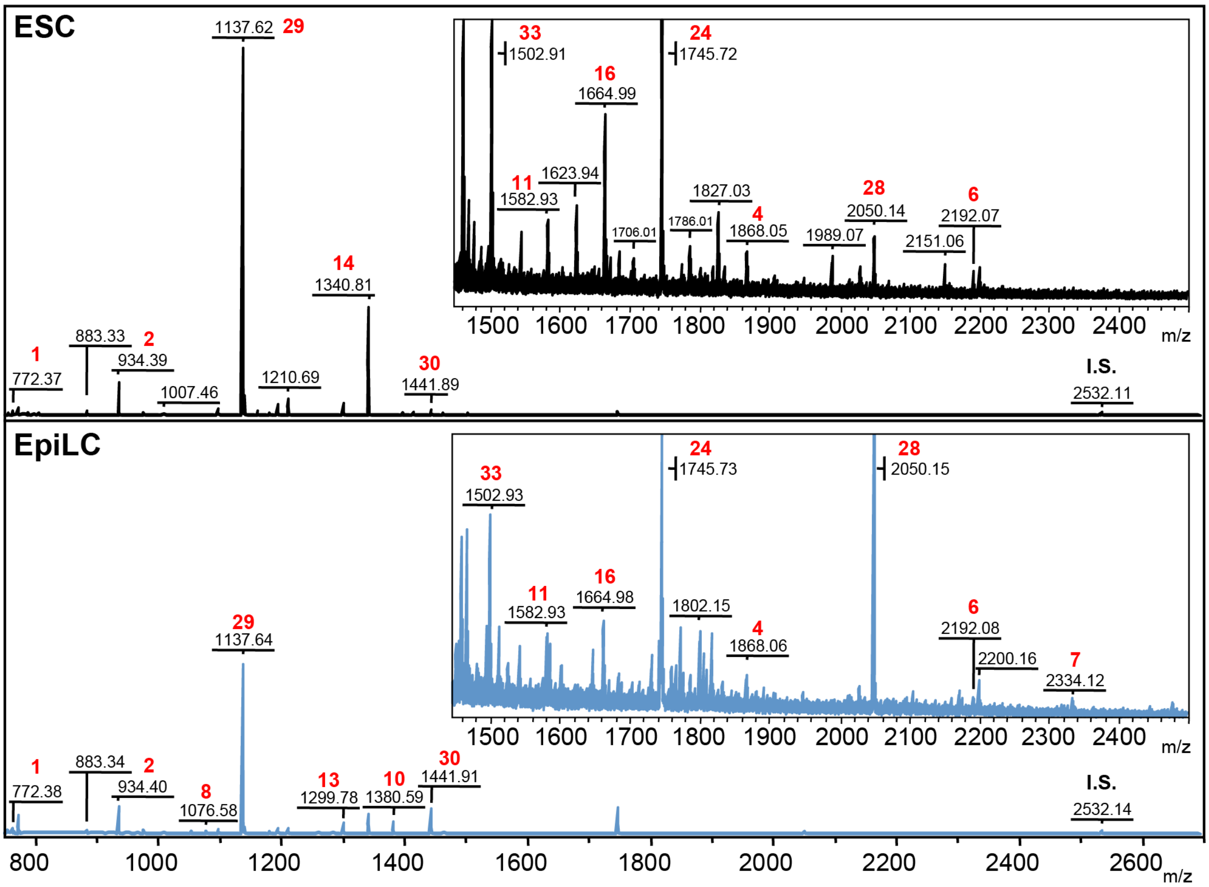

Supplementary Fig. S8 GSL structure quantification. Representative GSL MS spectra of ESCs (upper panel) and EpiLCs (lower panel). To allow intensity comparison across ESCs and EpiLCs, MS signals in ESC and EpiLC spectra are scaled to the same I.S. (internal standard) signal area. Estimated structures are numbered and, together with their absolute amount, listed in Supplementary Table S4.

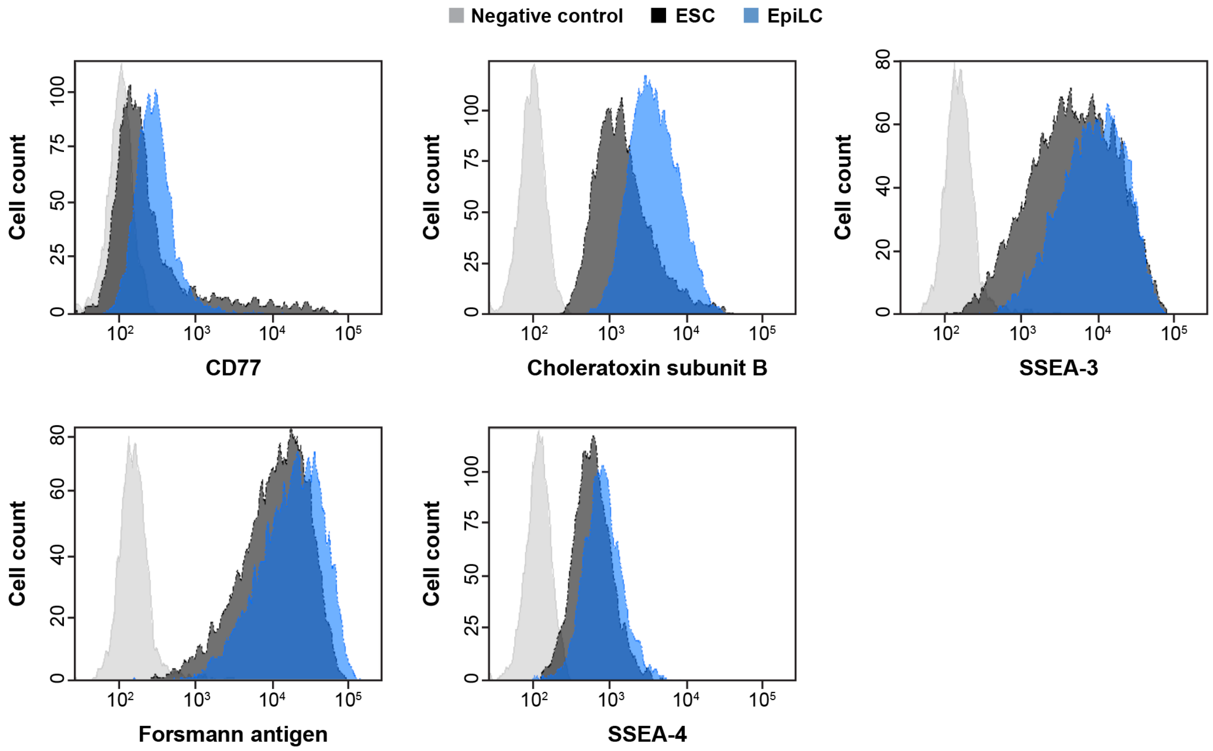

Supplementary Fig. S9 GSL structure profiling. Representative FACS histograms of ESCs and EpiLCs. Negative control: grey; ESC: black; EpiLC: blue.

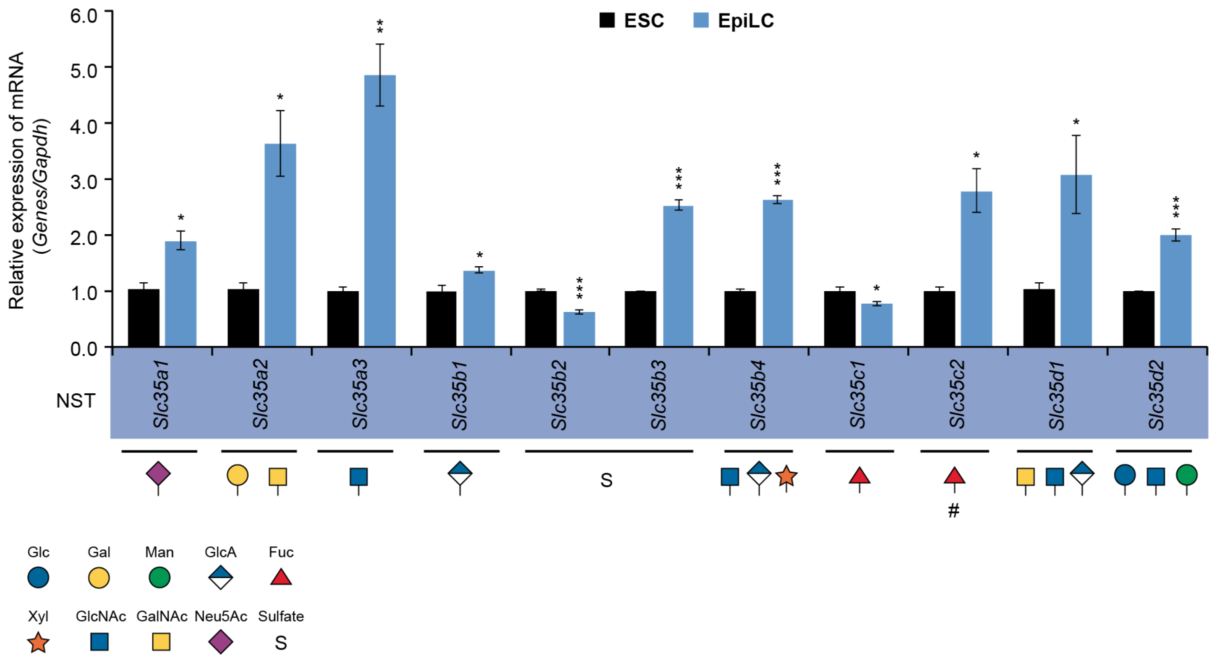

Supplementary Fig. S10 Nucleotide sugar transporter (NST) transcriptional analysis. Real-time PCR analysis of NST normalized against *Gapdh* in ESCs and EpiLCs, and shown as a fold change relative to ESCs. # denotes enzyme putative activity. Values are shown as means ± s.e.m. of three independent experiments. Significant values are indicated as * $P < 0.05$, ** $P < 0.01$, and *** $P < 0.001$.

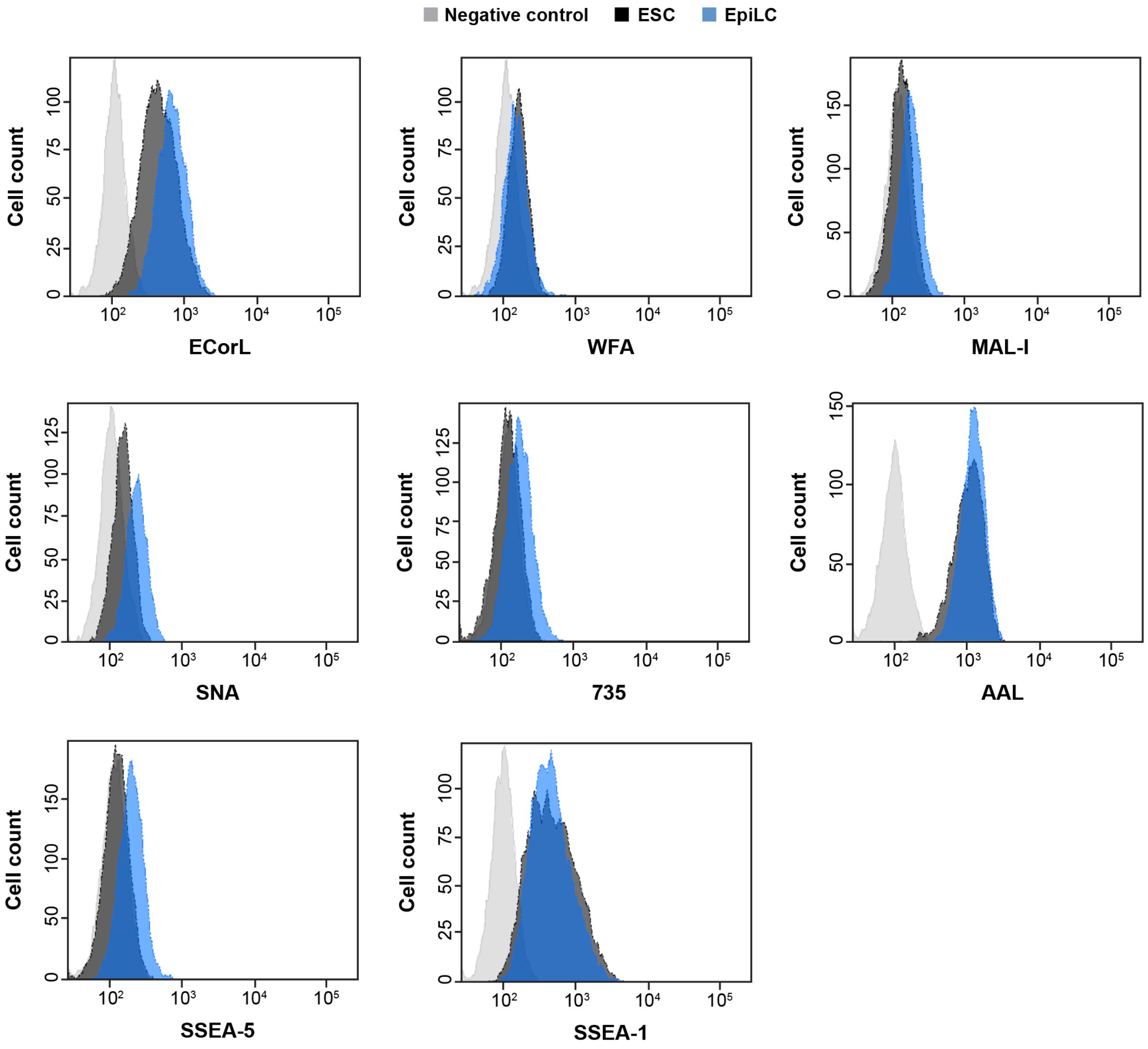

Supplementary Fig. S11 Pathway-non-specific structure profiling. Representative FACS histograms of ESCs and EpiLCs. Negative control: grey; ESC: black; EpiLC: blue.

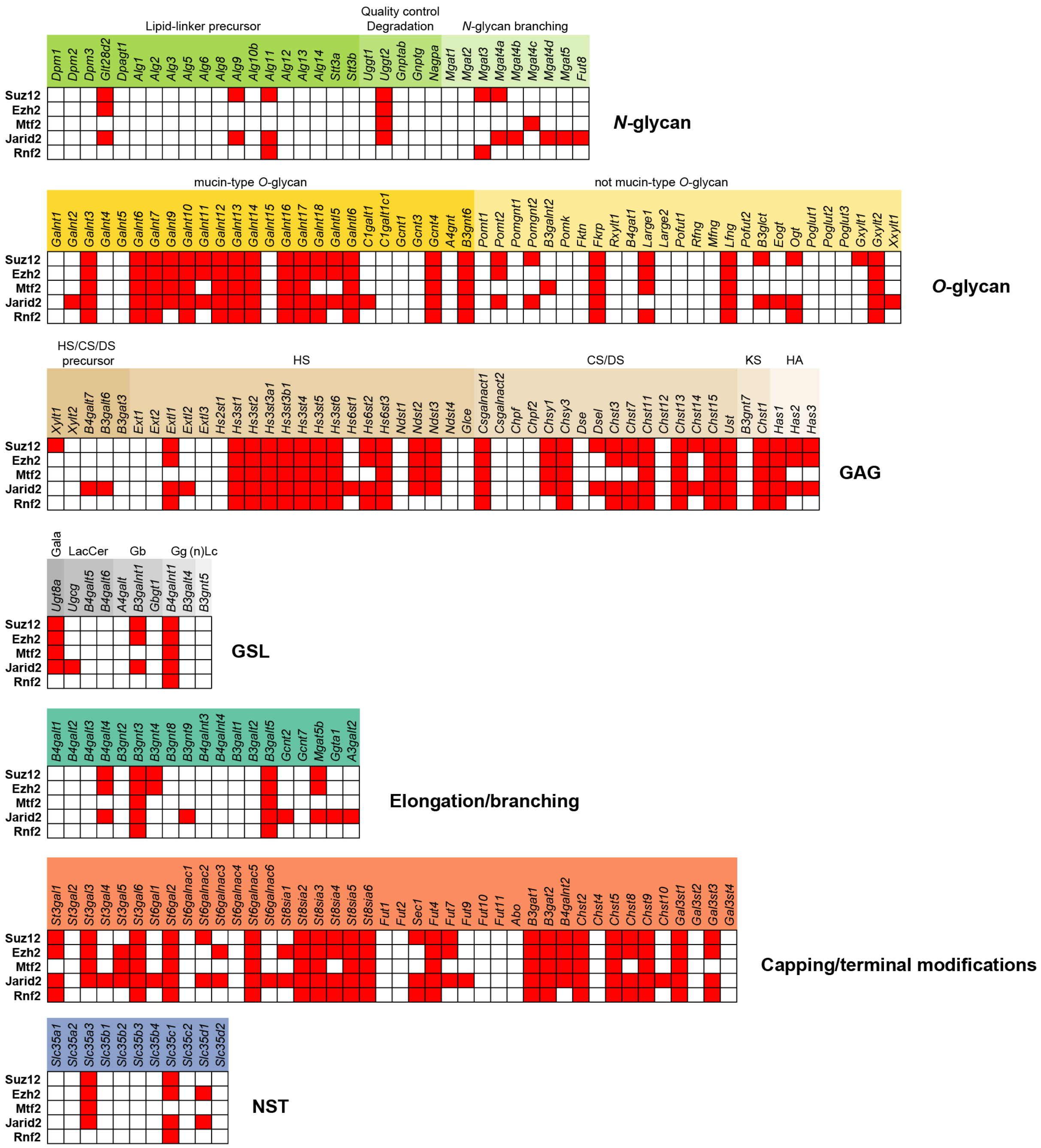

Supplementary Fig. S12 PRC components bind to glycosyltransferases genes. Analyzed ChIP-seq datasets were obtained from wild-type/untreated ESCs precipitated using: anti-Suz12 Ab (SRX1372665) (ref. 46), anti-Ezh2 Ab (SRX2528911) (ref. 47), anti-Mtf2 Ab (SRX2776968) (ref. 48), anti-Jarid2 Ab (SRX3738839) (ref. 47) and anti-Rnf2 Ab (SRX191072) (ref. 49). To identify factors enriched specifically in ESCs at the glycosyltransferase promoter regions, we used the “Enrichment analysis” option present on ChIP-Atlas database and we grouped ESC and EpiLC specific glycosyltransferases based on their expression. The ChIP

occupancy of the top resulting factors of the "Enrichment analysis", particularly enriched for PRC2 components, was then examined in detail at the promoter region of each glycosyltransferase. ChIP occupancy (red) was determined within a range of ± 5kb and with a threshold for statistical significance set as 50 (1 < 1E-05) calculated by peak-caller MACS2.

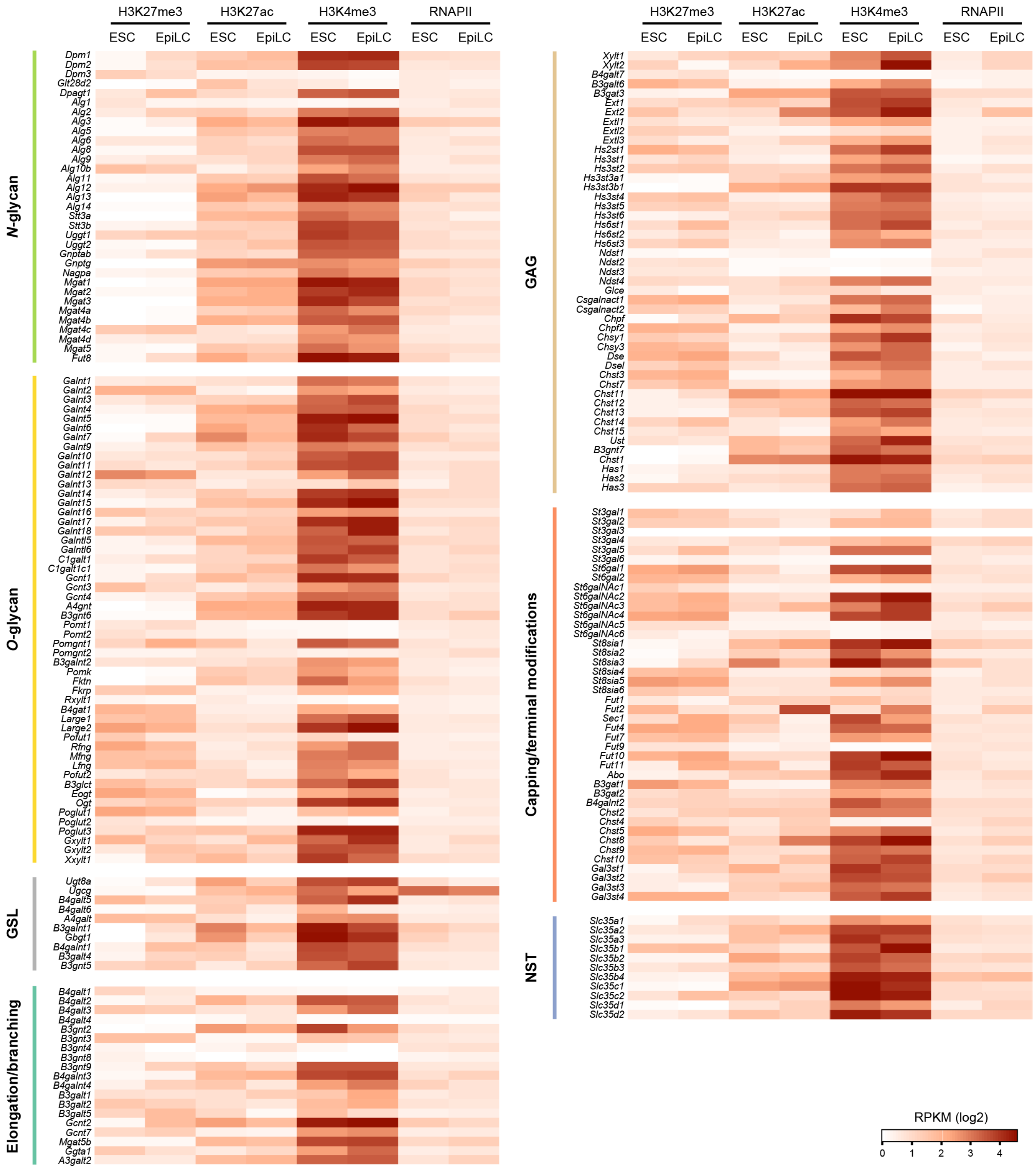

Supplementary Fig. S13 Epigenetic global alteration during ESC to EpiLC transition. Heatmap of ChIP-seq read density profiles for H3K27me3 (ESC: SRX4488301; EpiLC: SRX4488308) (transcription repression), H3K27ac (ESC: SRX4488293; EpiLC: SRX4488300) (transcription activation), H3K4me3 (ESC: SRX4488285; EpiLC: SRX4488292) (active promoter marker) and RNA polymerase II (RNAPII) (ESC: SRX4488317; EpiLC: SRX4488324) on glycosylation-related genes. ChIP-seq datasets were analyzed from ref. 7. ChIP-seq read density values are listed in Supplementary Table S1.

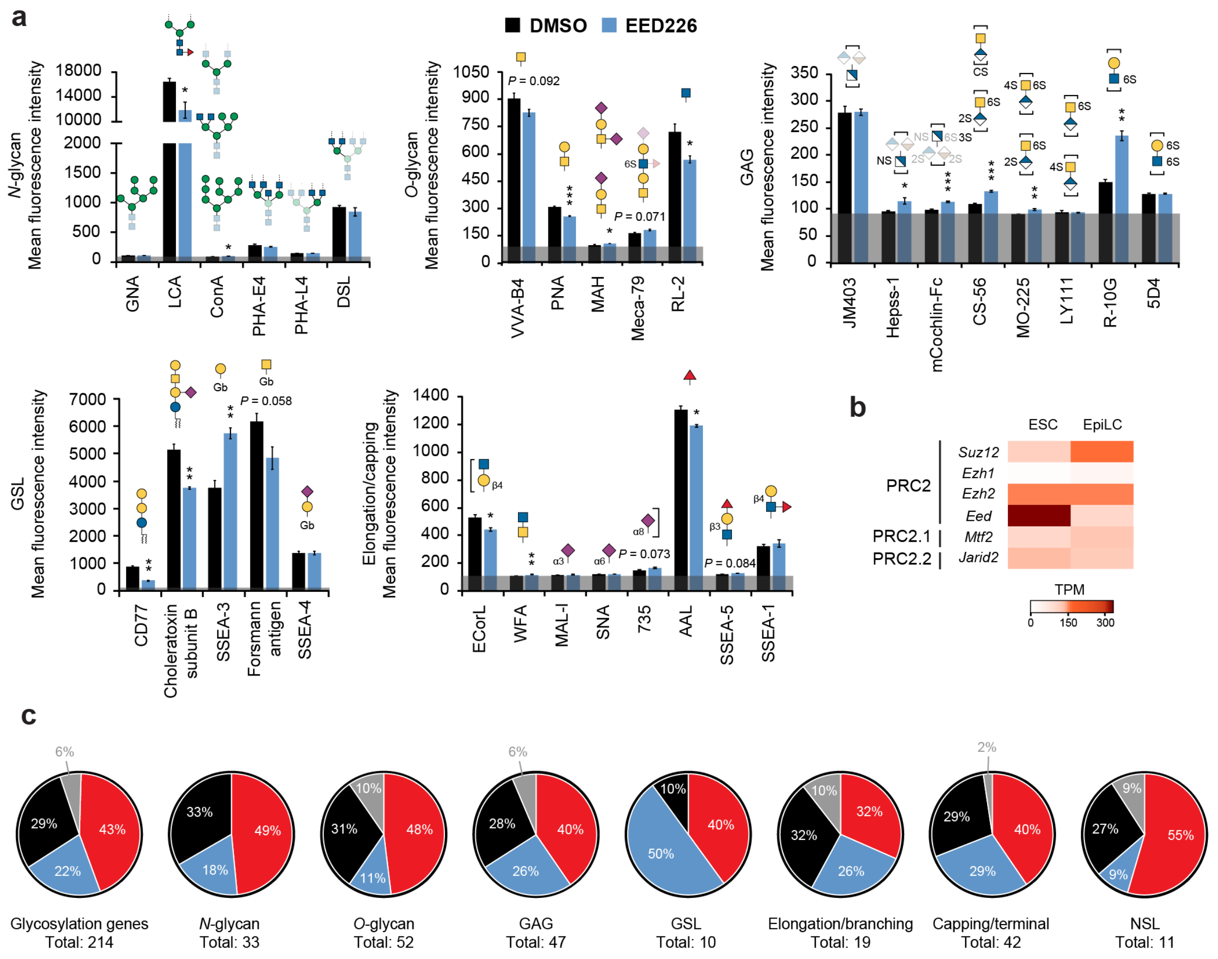

Supplementary Fig. S14 PRC2 regulates glycosylation in ESCs. **a** Glycosylation structure profiling by FACS using specific lectins/Abs in ESCs after EED226 treatment for 48h. Lectin/Ab specificities are schematically represented above each histogram. The grey line at the bottom represents the negative control staining. Values are shown as means ± s.e.m. of three independent experiments. Significant values are indicated as * $P < 0.05$, ** $P < 0.01$, and *** $P < 0.001$. **b** Heat map representing expression level of PRC2 core components and PRC2.1 and PRC2.2 main accessory subunits detected in ESCs and EpiLCs by RNA-seq. **c** Pie charts showing percentage of glycosylation related genes in each expression pattern by comparing ESCs/EpiLCs and EED226 untreated/EED226 treated samples expression by RNA-seq. Genes following a similar expression pattern during EpiLC differentiation and EED226 treatment (red), opposite patterns (blue), and changed in EpiLCs but not upon EED226 treatment (black) (the grey label indicates genes which expression was unchanged). RNA-seq data are obtained from a single experiment.

Supplementary Table S1

ChIP-seq read density values (RPKM) analyzed from ref. 7 relative to Supplementary Fig. S13.

Supplementary Table S2

List of primer sets used for Real-time PCR analysis.

Supplementary Table S3

List of primary and secondary Abs and lectins used with their respective specificity.

Supplementary Table S4

MS and HPLC detected structures and absolute amounts.

Supplementary Table S5

List of glycosyltransferases genes and related enzymes (TPM) divided per expression pattern relative to Supplementary Fig. S14c.

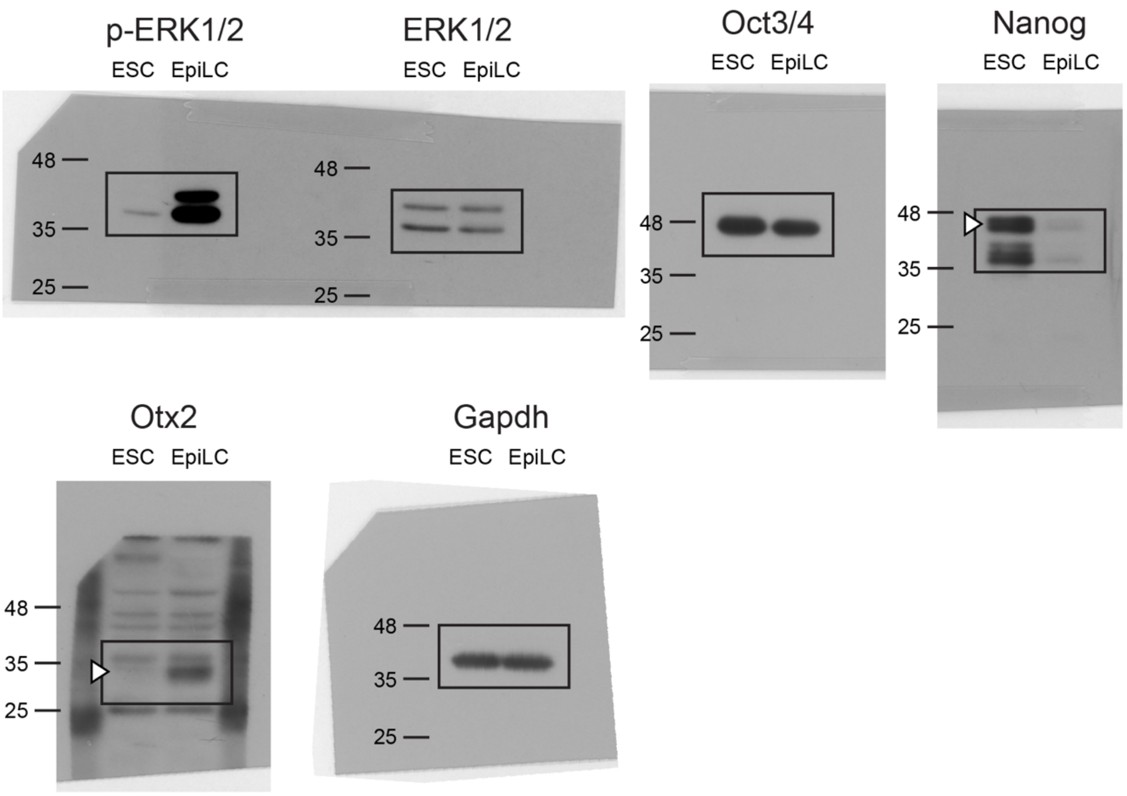

Supplementary Fig. S15 Uncropped gels/blots related to Fig. 1.

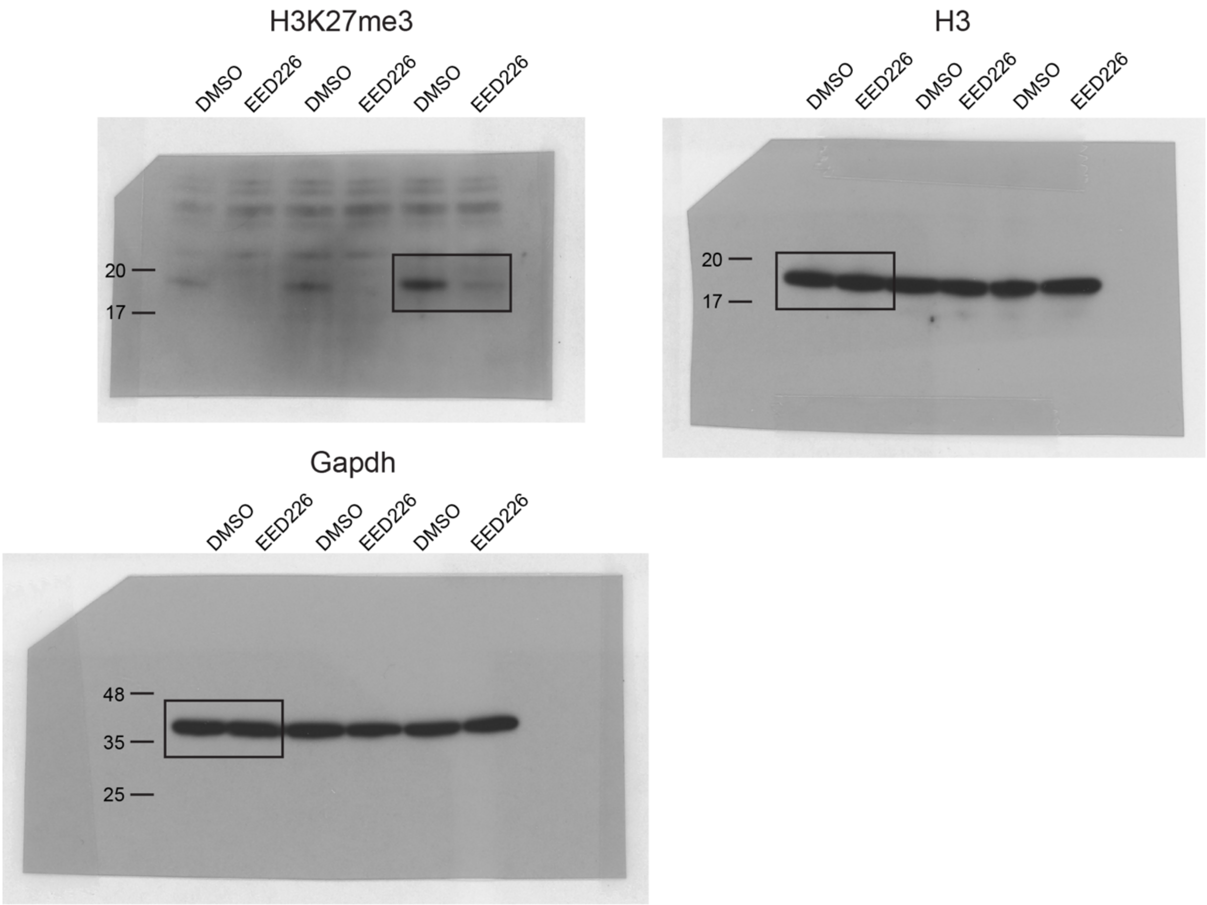

Supplementary Fig. S16 Uncropped gels/blots related to Fig. 7.
